# Supplementary material for: MicroRNA-31 Regulates Chemosensitivity in Malignant Pleural Mesothelioma
Source: Mol Ther Nucleic Acids. 2017 Jul 8;8:317–29. doi: 10.1016/j.omtn.2017.07.001 (PMC5537169; doi:10.1016/j.omtn.2017.07.001)
Supplement: Document S1. Figures S1–S12, and Table S1 [file mmc1.pdf]

OMTN, Volume *8*

Supplemental Information

MicroRNA-31 Regulates Chemosensitivity in Malignant Pleural Mesothelioma

Hannah L. Moody, Michael J. Lind, and Stephen G. Maher

A

Cell line	Mean miR-31 CT value	Mean RNU-6 CT value
NCI-H2452 parent	38.96061	19.04827
NCI-H2452 miR-VC	38.85159	18.65891
NCI-H2452 miR-31	27.66615	19.33311
P31	24.86754	19.42408
NCI-H2052	23.28798	19.36644
MSTO-211H	27.50758	23.85113

B

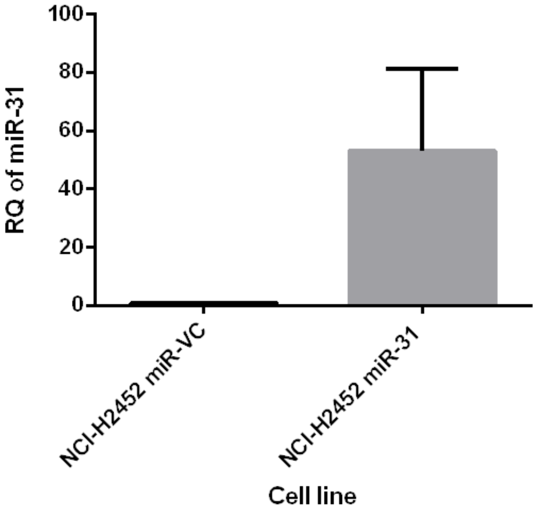

Supplementary Figure 1. MiR-31 status in MPM cell lines. (A) C_T values of MPM cell lines comparing miR-31 expression. (B) qPCR evaluating the relative level of expression miR-31 between vector control and miR-31 transfect cells over a period of 6 months (*n*=5). All qPCR runs were loaded with the maximum template of 100ng cDNA.

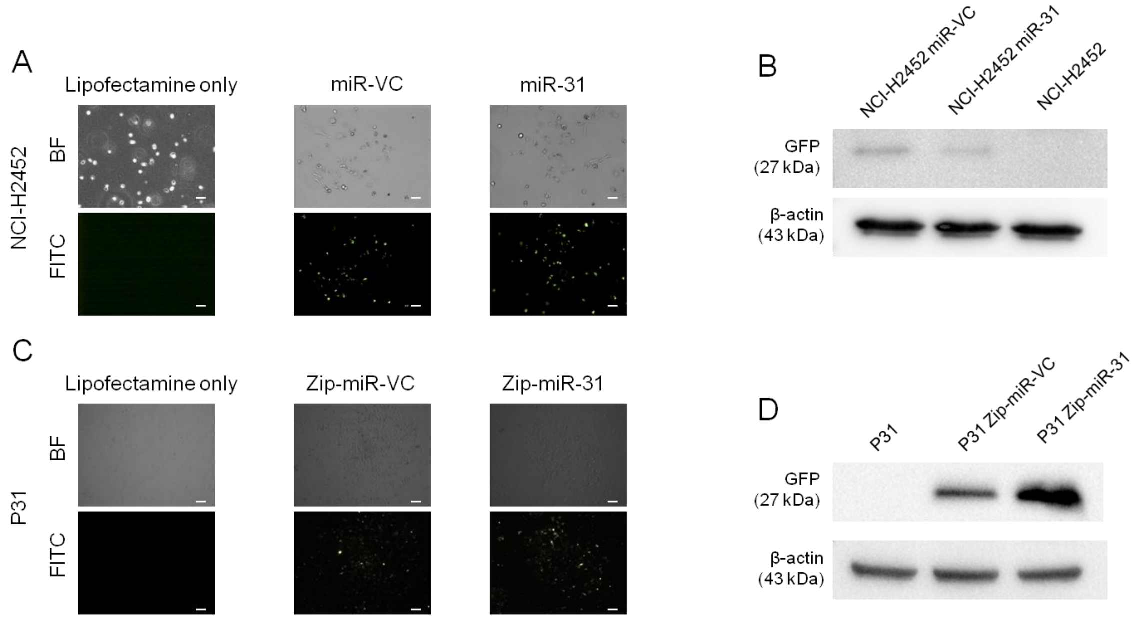

Supplementary Figure 2. Confirmation of stable transfection. (A) The NCI-H2452 cell line was stably transfected with corresponding overexpression vectors which had GFP reporter sequence. (B) Representative Western blot indicating GFP expression in transfected NCI-H2452 cell line. (C) The P31 cell line was stably transfected with corresponding silencing vectors which had GFP reporter sequence. (D) Representative Western blot indicating GFP expression in transfected P31 cell line. Images shown from FITC channel of Carl Zeiss Axio Vert.A1 microscope, captured with AxioCamIC with 0.5x Camera Adapter. The objective used was LD A-Plan 10x/0.25 Ph1.

A

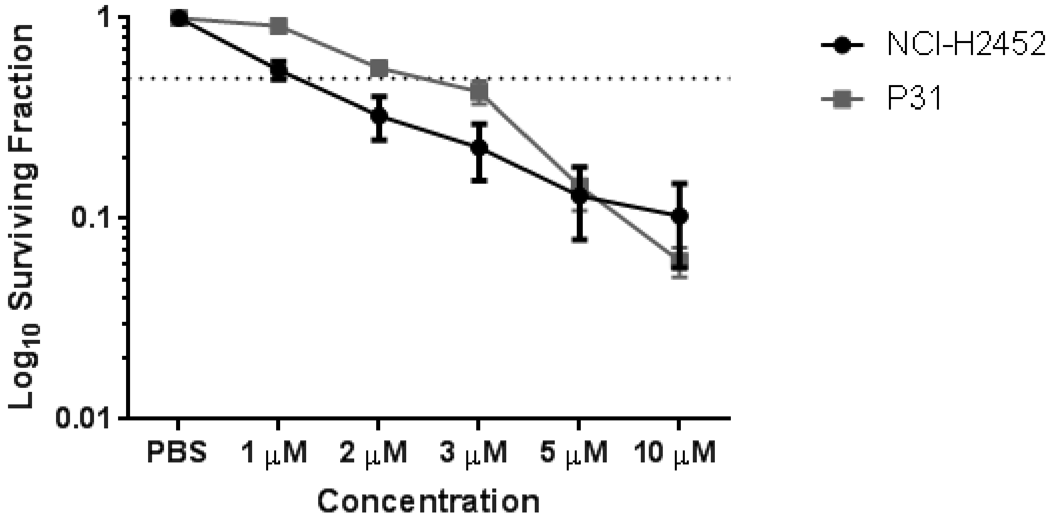

B

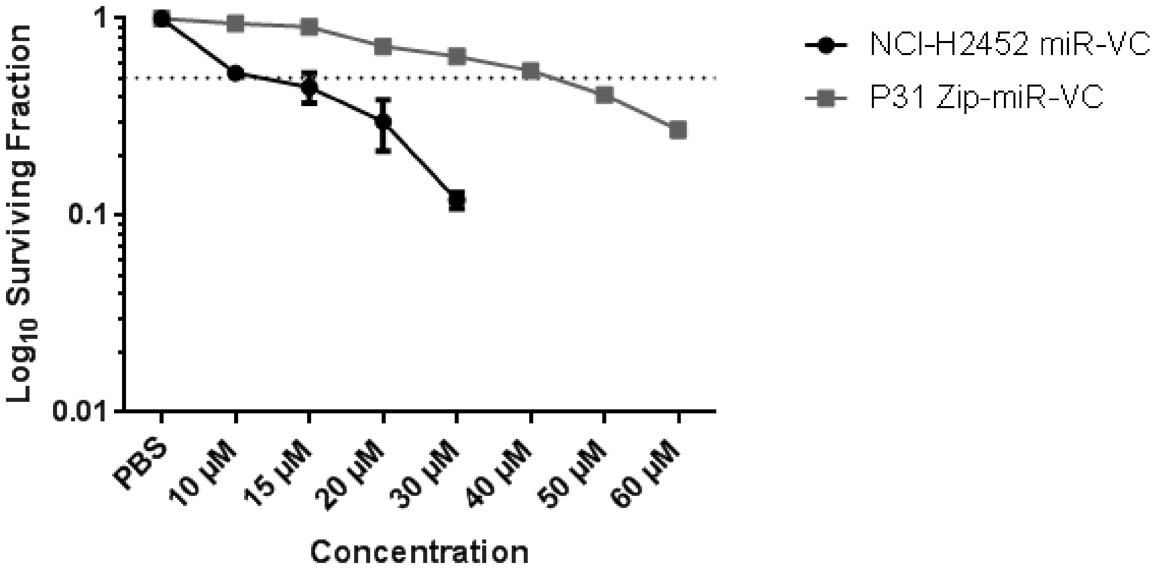

Supplementary Figure 3. IC_{50} doses of chemotherapeutics in MPM cell lines. (A) Dose response for cisplatin treatment for 24 h in the miR-31-deficient NCI-H2452 cell line (*n*=5) and P31 miR-31-positive cell line (*n*=3). Dose of 1 µM cisplatin determined for NCI-H2452, and 2 µM determined for P31. (B) Dose response for 48 h treatment with carboplatin in control transfected cell lines (*n*=3). Dose of 10 µM carboplatin determined for NCI-H2452 miR-VC, and 40 µM determined for P31 Zip-miR-VC. Cisplatin treatment was utilised for 24 h and carboplatin for 48 h in line with current literature, and due to more moderate effect of carboplatin treatment. All clonogenic assays were vehicle controlled with the analysis of PBS treated controls taken into account in surviving fraction calculations. Although plotted, where error bars are not visible, data replicates are within 0.05 surviving fraction.

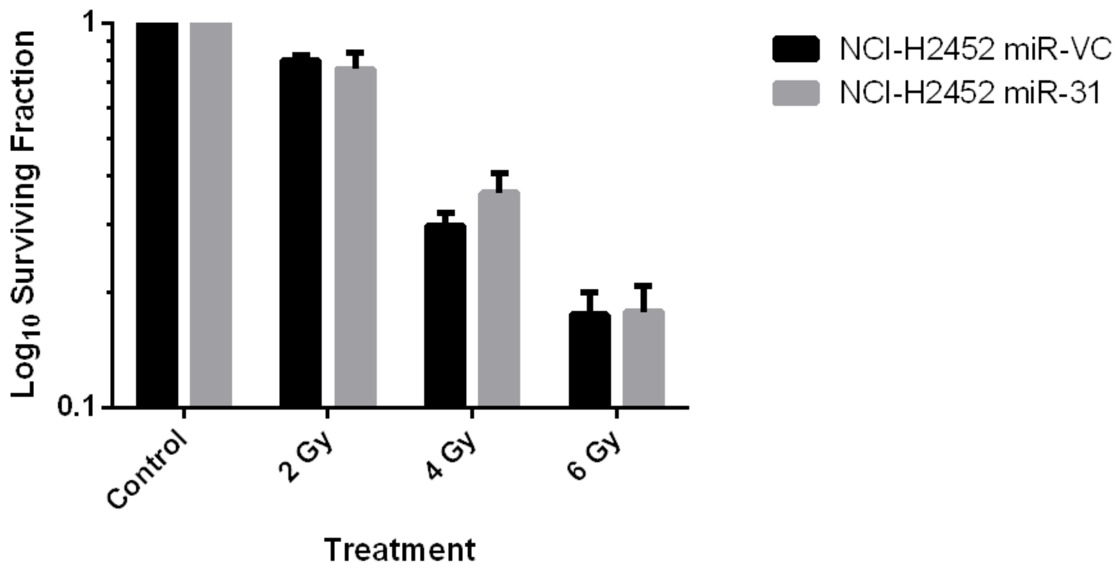

Supplementary Figure 4. The reintroduction of miR-31 does not modulate radiosensitivity in NCI-H2452. To determine the dose response for NCI-H2452 miR-VC and NCI-H2452 miR-31 cells, cells were irradiated with 2 Gy, 4 Gy and 6 Gy at a rate of 1.87 Gy/min. Survival was measured by clonogenic assay (*n*=3). Controls were mock irradiated. Data presented as ± SEM.

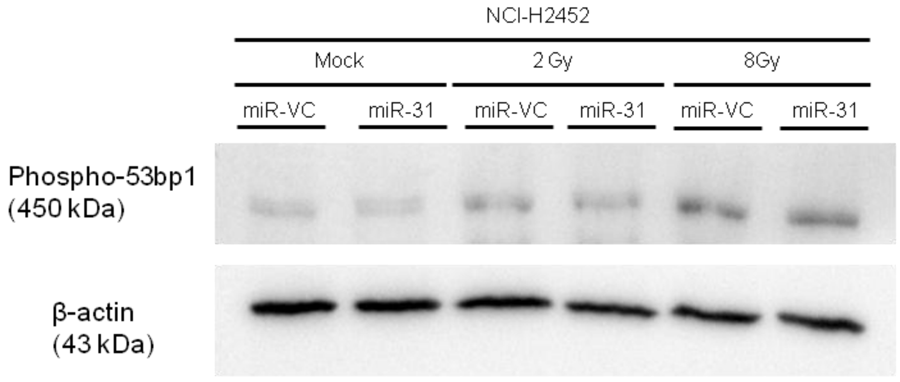

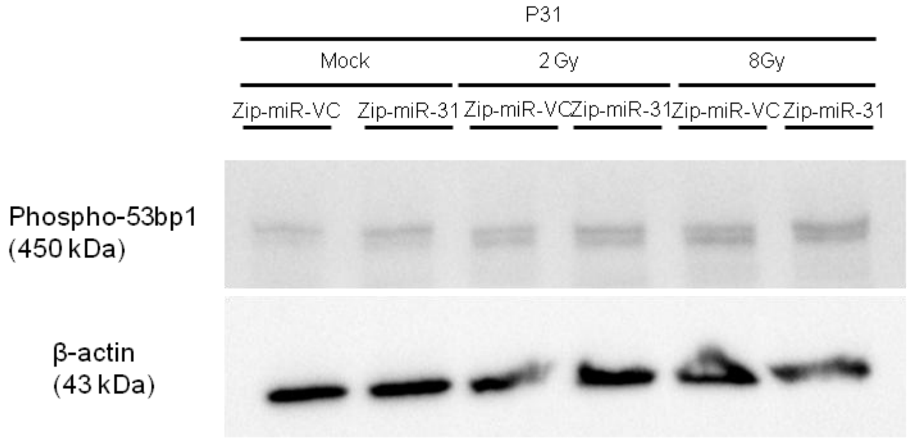

Supplementary Figure 5. MiR-31 does not modulate DNA damage induced by radiation treatment in MPM cells. Measuring phosphorylation of 53bp1 found no correlation between DNA damage induced by radiation treatment and miR-31 status. Cells were irradiated with 2 Gy and 8 Gy at a rate of 1.87 Gy/min. Controls were mock irradiated.

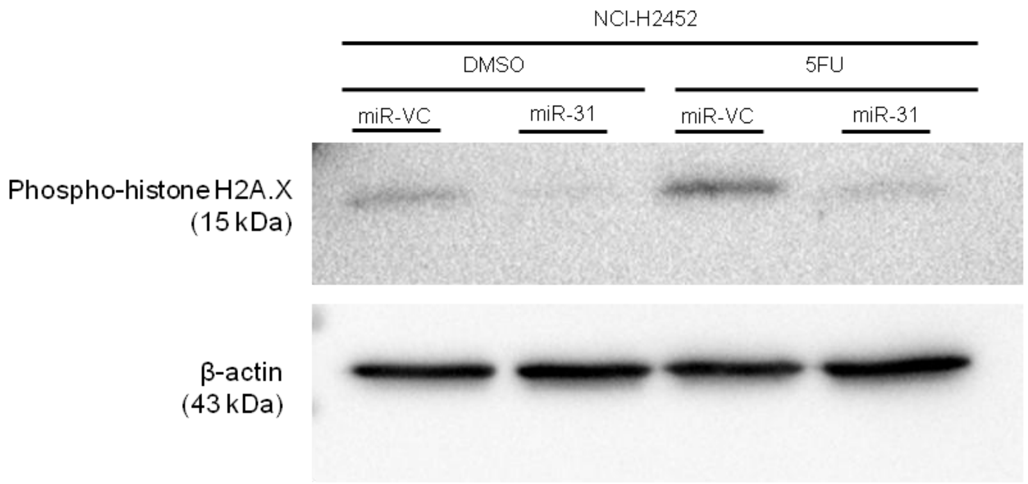

Supplementary Figure 6. Treatment with non-platinum based chemotherapeutics also decreases DNA damage. Treatment with 5FU induced minor DNA damage when miR-31 was reintroduced in the NCI-H2452 cell line, in accordance with cisplatin and carboplatin treatment. Cells were treated with 500µM 5FU for 24 hours.

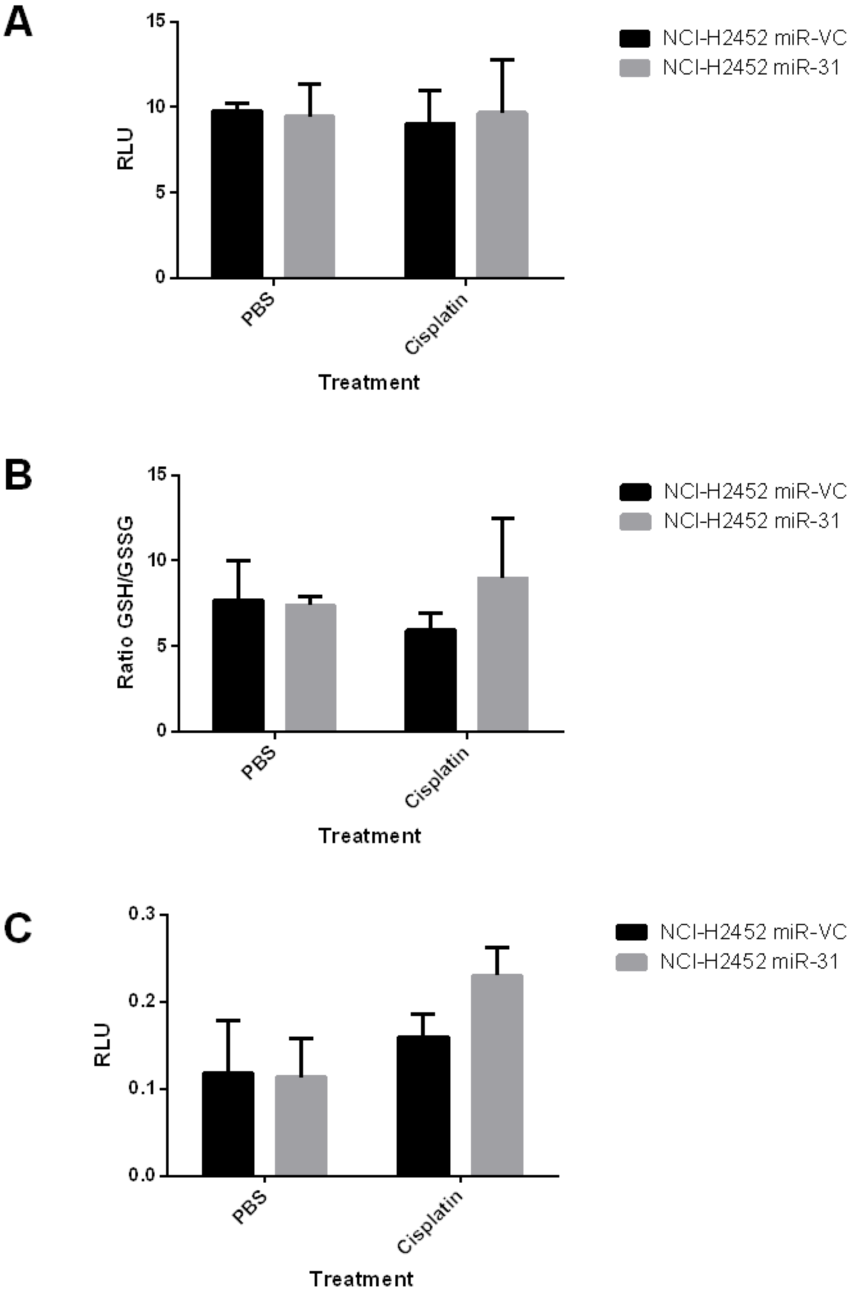

Supplementary Figure 7. Antioxidant and oxidant levels are negligibly altered by re-expression of miR-31. (A) Total glutathione levels were unaltered upon re-expression of miR-31, as measured by GSH/GSSG glo™ (Promega) assay (*n*=2). (B) Glutathione/Oxidised glutathione ratio was unaltered with miR-31 reintroduction, as measured by GSH/GSSG glo™ (Promega) assay (*n*=2). (C) Reactive oxygen species levels were moderately increased by re-expression of miR-31 with cisplatin treatment, as measured by ROS glo™ (Promega) assay (*n*=2). Data presented as ± SEM.

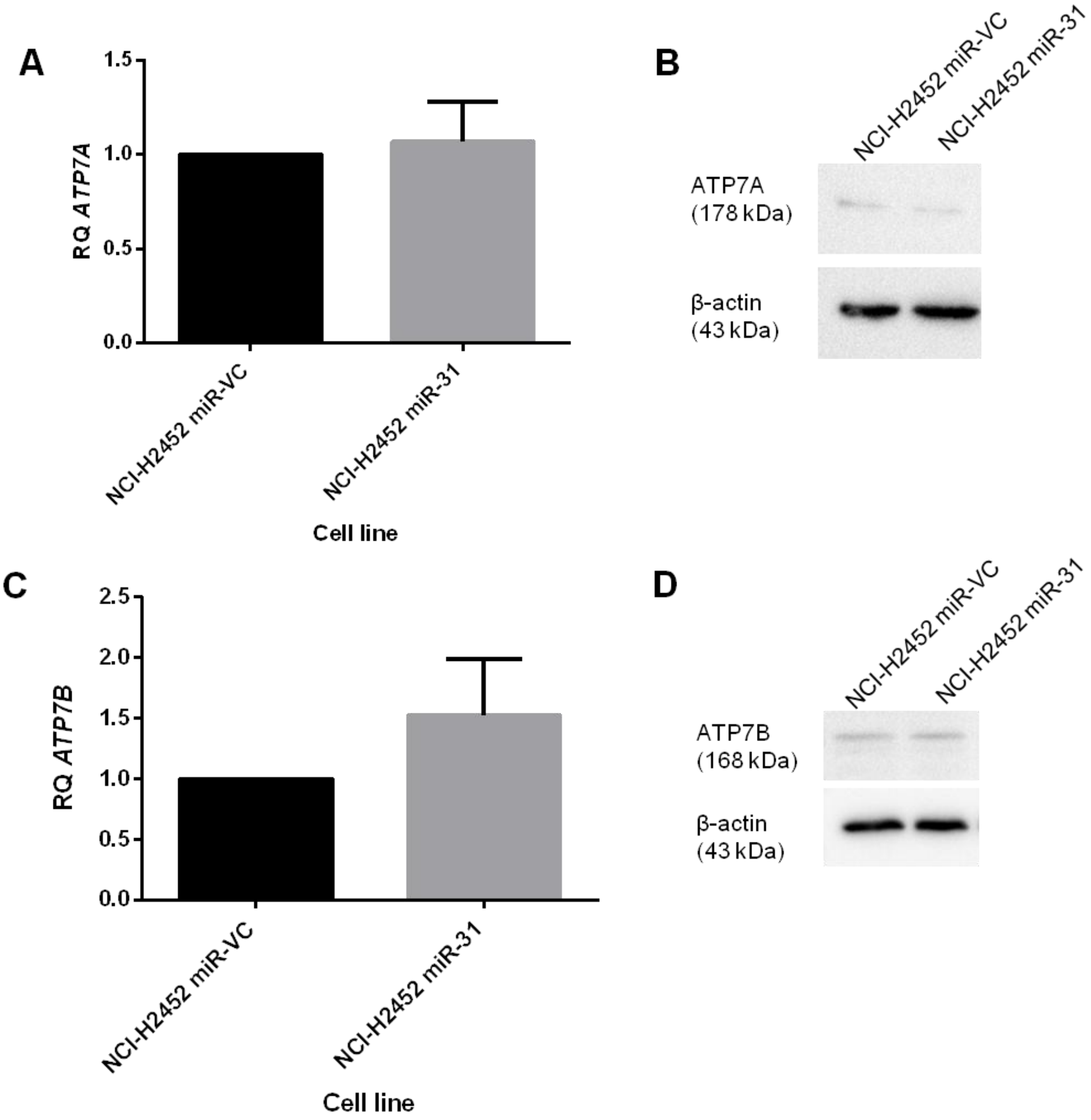

Supplementary Figure 8. MiR-31 rehabilitation in NCI-H2452 cells does not significantly alter the expression of copper efflux transporters. (A) Although a trend is apparent, there is no significant relative expression level of the transporter *ATP7A* in miR-31 compared to miR-VC equivalent (*n*=2). (B) ATP7A protein levels appear to be unaltered by miR-31 reintroduction. Data presented as the mean ± SEM. (C) Although a trend is apparent, there is no significant relative expression level of the transporter *ATP7B* in miR-31 compared to miR-VC equivalent (*n*=4). (D) ATP7B protein levels appear to be unaltered by miR-31 reintroduction. Data presented as the mean ± SEM.

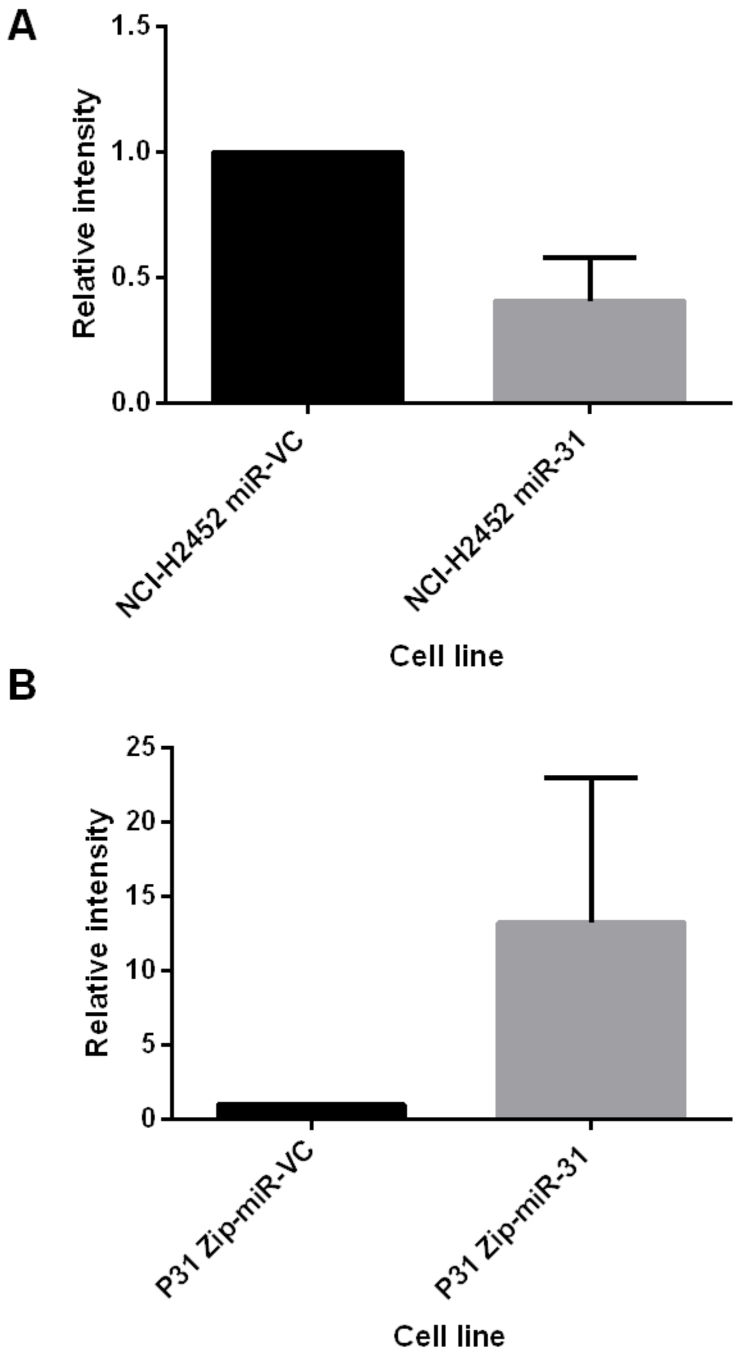

Supplementary Figure 9. Densitometry analysis for phospho-histone H2A.X. (A) There is a downregulation in the DNA damage marker phospho-histone H2A.X with miR-31 reintroduction, and an upregulation (B) with miR-31 suppression (*n*=3). Data presented as ± SEM.

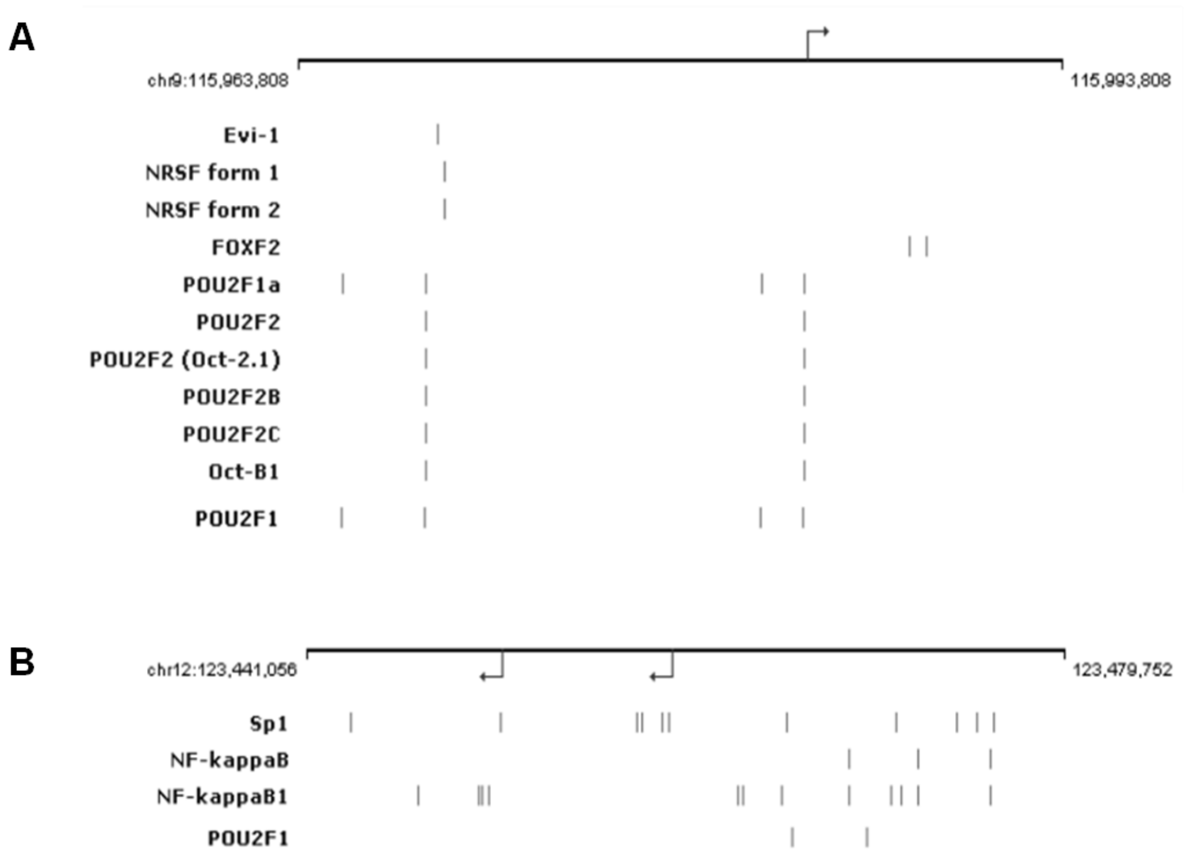

Supplementary Figure 10. Potential transcriptional regulators of genes *slc31a1* (CTR1) and *abcb9* (ABCB9). (A) Transcription factors regulating gene *slc31a1*. The image displays transcription factor binding sites in this gene promoter as predicted by SABiosciences' Text Mining Application and the UCSC Genome Browser. The *pou2f1* gene relates to the bipotential transcription factor OCT1. (B) Transcription factors regulating gene *abcb9*. The image displays transcription factor binding sites in this gene promoter as predicted by SABiosciences' Text Mining Application and the UCSC Genome Browser. The *pou2f1* gene relates to the bipotential transcription factor OCT1. (Adapted from http://www.sabiosciences.com/chipqpcrsearch.php).

Supplementary Table. 1. Target prediction for OCT1. Utilising bioinformatic tools, pairing of miR-31 with the *pou2f1* (OCT1) gene was performed. Of the approximately 190 predicted miRNA that may potentially bind to OCT1, miR-31 appears to be one of the most conserved. (Adapted from http://mircode.org/?gene=pou2f1&mirfam=&class=&cons=&trregion=, accessed 290615 and http://www.targetscan.org/cgibin/targetscan/vert_61/view_gene.cgi?taxid=9606&rs=NM_001198783&members=&showcnc=0&shownc=0&showncf=#miR-31, accessed 290615).

<table>
<tr><th rowspan="2">microRNA family</th><th rowspan="2">Predicted consequential pairing of target region (top) and miRNA (bottom)</th><th rowspan="2">Seed position</th><th rowspan="2">Transcript region</th><th colspan="3">Conservation</th></tr>
<tr><th>Primates</th><th>Mammals</th><th>Other vertebrates</th></tr>
<tr><td>miR-31</td><td>5' ...UUCUUAGUGAGAUCAUCUUGCCA..
| | | | | | |
3' UCGAUACGGUCGUAGAACGGA</td><td>chr1:167389158</td><td>3pUTR</td><td>78%</td><td>61%</td><td>0%</td></tr>
<tr><td>miR-31</td><td>5' ...UUUUUCUUAUAUCUGUCUUGCCA...
| | | | | | |
3' UCGAUACGGUCGUAGAACGGA</td><td>chr1: 167391236</td><td>3pUTR</td><td>100%</td><td>65%</td><td>23%</td></tr>
</table>

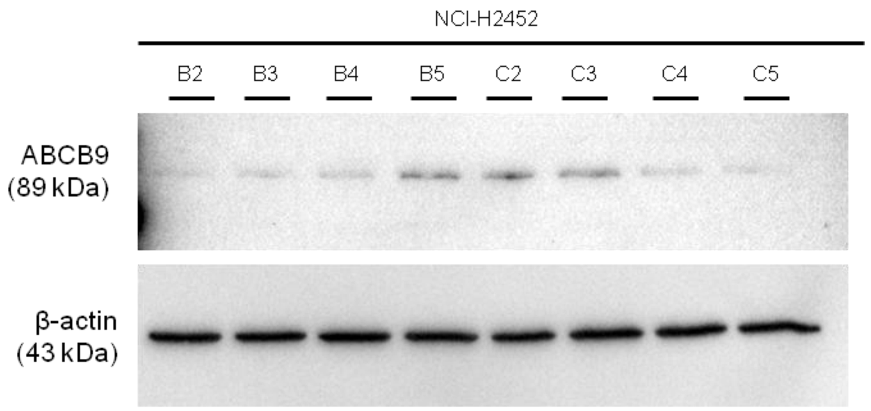

Supplementary Figure 11. ABCB9 overexpression is stable selected clones. NCI-H2452 miR-31-null cell line was transfected with EX-NEG vector control plasmid and an ABCB9 overexpression plasmid. Clonal populations were selected and grown under puromycin selection. Clones B2, B3, B4 and B5 correspond to EX-NEG vector control populations. Clones C2, C3, C4, C5 correspond to ABCB9 overexpressing clones. Clones C2 and C3 were selected as high expressing clones, C4 and C5 classified as low ABCB9 expressing clones.

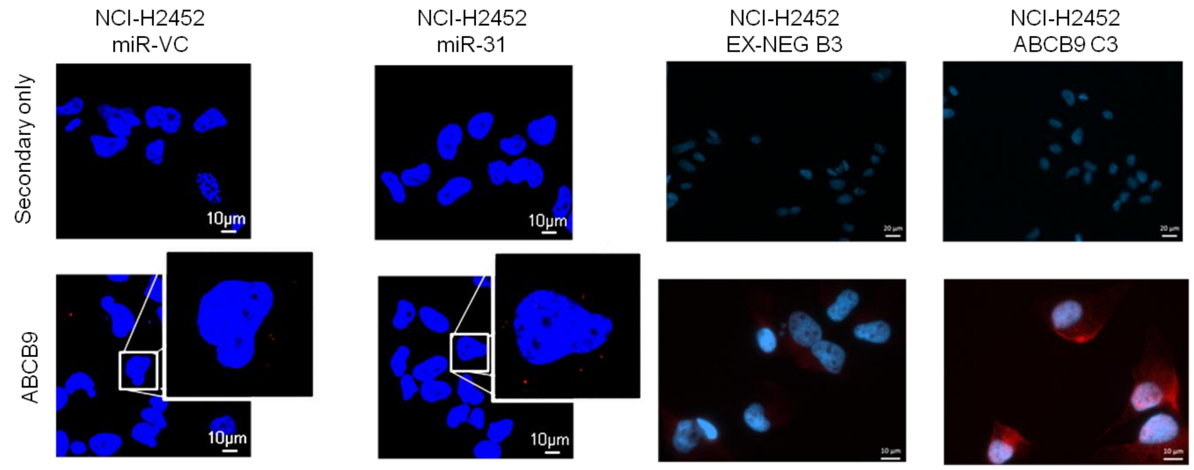

Supplementary Figure 12. ABCB9 overexpression in miR-31 reintroduced versus NCI-H2452 clonal variants. Immunoflourescent images showing overexpression of ABCB9 in transfected cells. ABCB9 staining (red), nuclei stained with DAPI (blue). Images captured on Zeiss Axio Vert.A1 with N-Achroplan 100x objective. Images for both EX-NEG and ABCB9 overexpressing populations are set at the same exposure in the same channels.
