# Supplementary material for: MicroRNA-31 Regulates Chemosensitivity in Malignant Pleural Mesothelioma
Source: Mol Ther Nucleic Acids. 2017 Jul 8;8:317–29. doi: 10.1016/j.omtn.2017.07.001 (PMC5537169; doi:10.1016/j.omtn.2017.07.001)
Supplement: Document S2. Article plus Supplemental Information [file mmc2.pdf]

Molecular Therapy
Nucleic Acids
Original Article

MicroRNA-31 Regulates Chemosensitivity in Malignant Pleural Mesothelioma

Hannah L. Moody,[1,2] Michael J. Lind,[2,3] and Stephen G. Maher[1,4]

[1]School of Life Sciences, University of Hull, Hull HU6 7RX, UK; [2]Hull York Medical School, Hull HU6 7RX, UK; [3]Centre for Oncology and Haematology, Castle Hill Hospital, Hull and East Yorkshire NHS Trust, Cottingham HU16 5JQ, UK; [4]Trinity Translational Medicine Institute, Department of Surgery, Trinity College Dublin, Dublin 8, Ireland

Malignant pleural mesothelioma (MPM) is associated with an extremely poor prognosis, and most patients initially are or rapidly become unresponsive to platinum-based chemotherapy. MicroRNA-31 (miR-31) is encoded on a genomic fragile site, 9p21.3, which is reportedly lost in many MPM tumors. Based on previous findings in a variety of other cancers, we hypothesized that miR-31 alters chemosensitivity and that miR-31 reconstitution may influence sensitivity to chemotherapeutics in MPM. Reintroduction of miR-31 into miR-31 null NCI-H2452 cells significantly enhanced clonogenic resistance to cisplatin and carboplatin. Although miR-31 re-expression increased chemoresistance, paradoxically, a higher relative intracellular accumulation of platinum was detected. This was coupled to a significantly decreased intranuclear concentration of platinum. Linked with a downregulation of OCT1, a bipotential transcriptional regulator with multiple miR-31 target binding sites, we subsequently identified an indirect miR-31-mediated upregulation of ABCB9, a transporter associated with drug accumulation in lysosomes, and increased uptake of platinum to lysosomes. However, when overexpressed directly, ABCB9 promoted cellular chemosensitivity, suggesting that miR-31 promotes chemoresistance largely via an ABCB9-independent mechanism. Overall, our data suggest that miR-31 loss from MPM tumors promotes chemosensitivity and may be prognostically beneficial in the context of therapeutic sensitivity.

INTRODUCTION

Malignant mesothelioma (MM) is a relatively rare, aggressive tumor that originates in the cells that compose the mesothelial surface of coelomic cavities of the body. The pleural form of malignant mesothelioma (MPM) is the most frequent presentation of the disease, with other areas of the body contributing to approximately 22% of MM cases.[1–3] The primary agent for MPM pathogenesis is exposure to asbestos fibers.[4] Unfortunately, the disease has a long latency period with onset between 20 and 40 years after exposure. Patients diagnosed with MPM are faced with an extremely poor prognosis (median survival < 9 months after diagnosis), which is mainly attributed to poor responses to platinum-based chemotherapeutics, the primary treatment modality for MPM, alongside the anti-folate pemetrexed.[5,6] Acquisition and maintenance of chemotherapy-resistant phenotypes remain both clinical and scientific challenges in MPM. Therefore, investigation into the affected molecular pathways and how these are regulated is of high importance in improving the prognosis and survival times in patients who have limited options.

MicroRNAs (miRNAs) are small, non-coding RNAs approximately 18–22 nucleotides in length that function to regulate gene expression at the post-transcriptional level[7] (comprehensively reviewed in Lynam-Lennon et al.[8] and Maher et al.[9]). miRNAs are involved in all fundamental biological pathways and cellular processes, so it is not surprising that miRNA dysfunction is considered instrumental in the acquisition of the hallmarks of cancer.[10] miRNAs are novel therapeutic targets and promising biomarkers with potential applications in diagnosis, prognosis, tumor staging, predicting patient response to treatment, and determining developmental lineages and clinical subtypes.[11–14]

The chromosomal fragile site at 9p21.3 is often lost in mesotheliomas and encodes p16, a potent tumor suppressor and cell-cycle regulator.[15,16] In addition, the fragile site encodes microRNA-31 (miR-31), making this miRNA one of the most commonly deleted in MPM. Ivanov et al.[17] previously reported a downregulation of miR-31 due to deletions on chromosome 9p21.3 in approximately 54% of tumors from MPM patients. It was observed that the reconstitution of miR-31 inhibited factors involved in cell-cycle progression and DNA repair, in line with our previous findings in esophageal adenocarcinoma.[18] In contrast, miR-31 downregulation has been correlated with increased long-term survival in patients, with the proposition that miR-31 can act as an oncogenic microRNA in MPM.[19] The research indicated a significant negative relationship between miR-31 expression and survival time in an aggressive sarcomatoid MPM patient cohort.[19] Studies have indicated, as in other cancers,[20,21] that miRNAs play pivotal roles in the onset and progression of MPM, making them putative targets for therapy or markers of prognosis.

Given our previous finding that miR-31 regulates DNA repair gene expression in esophageal adenocarcinoma,[18] which has a similar etiology to MPM, and considering that there are supportive studies in

Received 23 November 2016; accepted 3 July 2017;
http://dx.doi.org/10.1016/j.omtn.2017.07.001.

Correspondence: Stephen G. Maher, Trinity Translational Medicine Institute, Rm 1.20, Trinity Centre for Health Sciences, Trinity College Dublin, St. James's Hospital, Dublin 8, Ireland.
E-mail: maherst@tcd.ie

This is an open access article under the CC BY license (http://creativecommons.org/licenses/by/4.0/).

MPM,[17] we hypothesized that miR-31 loss may promote resistance to chemotherapy via alterations in DNA damage and repair. Surprisingly, upon re-expression of miR-31 in an otherwise-deficient cell line, we observed increased resistance to *cis*-dichlorodiamineplatinum(II) (cisplatin), with the converse observed upon miR-31 suppression in an endogenously expressing MPM cell line, P31. We determined a significantly decreased platinum content within the intranuclear region of miR-31-expressing cells and found DNA damage induction to be lower upon miR-31 reintroduction. Finally, a potential negative regulator of transcription with multiple miR-31 binding sites in the 3′ UTR region, OCT1,[22] may promote the upregulation of lysosomal drug transport via ABCB9, implying the sequestration of cisplatin into intracellular compartments, whereby it cannot access the nuclear compartment to mediate its primary cytotoxic effects.[23,24] This mechanism may be used to evade DNA damage, promoting cellular resistance to platinum-containing chemotherapeutics. However, although the drug transporter ABCB9 was upregulated with miR-31 reintroduction, when ABCB9 was independently overexpressed in the miR-31 null NCI-H2452 cell line, there was a marked increase in sensitivity to cisplatin treatment. While this demonstrates a functional role for ABCB9 in modulating chemosensitivity, the data ultimately support that miR-31 promotes chemoresistance in MPM via an ABCB9-independent mechanism. Our data suggest that while deletions in chromosome 9p21.3 may be associated with an overall poor prognosis, the specific loss of miR-31 from this region may not contribute to the chemoresistance observed in MPM patients.

RESULTS

miR-31 Modulates Sensitivity to Platinum-Based Chemotherapy

Because miR-31 has previously been associated with resistance to therapy, the effect of either reintroducing or suppressing miR-31 in MPM cells was assessed. The miR-31 null epithelioid NCI-H2452 cell line was stably transfected with either miR-VC (microRNA vector control) or miR-31 expression plasmids, while epithelioid P31 cells expressing endogenous miR-31 were stably transfected with suppression plasmids Zip-miR-VC or Zip-miR-31 (Figures S1 and S2). To establish whether miR-31 modulation altered the overall sensitivity of cells treated with platinum-based chemotherapy, the clonogenic assay was applied using the appropriate inhibitory concentration 50 (IC_{50}) doses (Figure S3). The established IC_{50} doses were in line with previously published doses for these cell lines.[25–27] Higher doses of chemotherapies were used in further experiments to facilitate the study of mechanistic properties. To ascertain overall alterations in survival with treatment, the gold standard clonogenic assay was again used. There was a significant increase in the surviving fraction of 9.6 ± 1.6 in response to miR-31 reintroduction in NCI-H2452 with cisplatin treatment and a 19.3 ± 0.2 increase in the surviving fraction with *cis*-diammine(1,1-cyclobutanedicarboxylato)platinum(II) (carboplatin) treatment. Furthermore, there was an 8.4 ± 0.2 significant increase in the surviving fraction with miR-31 suppression, illustrating sensitivity to cisplatin upon suppression of miR-31 in the P31 cell line (Figure 1A). However, the silencing of miR-31 in the P31 cell line led to no significant difference in the surviving fraction when treated with carboplatin.

Following from the observation that reintroduction of miR-31 increased clonogenic survival in NCI-H2452 and decreased survival in the miR-31-suppressed P31 cell lines, a cumulative cell count was undertaken to first determine whether miR-31 alone, without the influence of chemotherapy, would alter proliferation, as previously noted in Ivanov et al.[17] In addition, the cumulative cell count attempted to ascertain at what time point miR-31 might influence proliferation. miR-31 manipulation without the influence of chemotherapy produced no significant change in proliferation rate; the cells only responded differently after cisplatin treatment, suggesting miR-31 plays an active role in the response to chemotherapy. A disparity in the ability of the miR-31-overexpressing NCI-H2452 cells to respond to cisplatin was observed, as evident in the comparison between treated groups in Figure 1B. A significant change in proliferation of NCI-H2452 miR-31 occurred only 9 days after treatment with cisplatin, whereas the vector control equivalent was significantly affected by cisplatin 3 days after treatment. Basal proliferation remained unaltered by miR-31 status, suggesting that with miR-31, cells had a delay in cytotoxic response (Figure 1B). While miR-31 modulates chemosensitivity, in order to assess whether the alterations in resistance were attributed specifically to chemotherapy, radiosensitivity was analyzed, which remained unaltered by miR-31 status in MPM cells (Figures S4 and S5). Although chemotherapy and radiotherapy both target DNA, the two modalities have considerably different mechanisms of action, indicating a chemotherapy-specific enhancement of resistance, most likely attributed to a mechanism that is chemotherapy limited.

miR-31 Alters Intracellular Distribution of Cisplatin in MPM

To begin to establish a potential mechanism underpinning miR-31-mediated chemoresistance, the influence of miR-31 on DNA damage induction was assessed via levels of phospho-histone H2A.X (γH2A.X), which is phosphorylated on serine 139 in response to DNA damage.[28] NCI-H2452 miR-31 cells displayed a reduction in γH2A.X levels, whereas P31 Zip-miR-31 cells demonstrated an increase in γH2A.X, both in response to cisplatin and carboplatin treatment (Figure 2). This suggests a role for miR-31 in either antagonizing DNA damage induction or promoting repair. The alteration to γH2A.X was not limited to platinum-based therapy; a similar trend was apparent upon treatment with 5-fluorouracil (5-FU), which has a different mechanism of action but still relies upon active transport into the intracellular environment (Figure S6). Differential activity of γH2A.X was also observed in response to DMSO treatment with miR-31 reintroduction. To determine whether this was due to gross alterations in DNA damage and repair pathways, levels of phospho-53bp1 were analyzed after irradiation treatment (Figure S5). No change was observed in radiation-treated groups, suggesting reliance upon altered transport and accumulation within the intracellular environment in miR-31-positive cells, rather than alterations in DNA damage induction or repair.

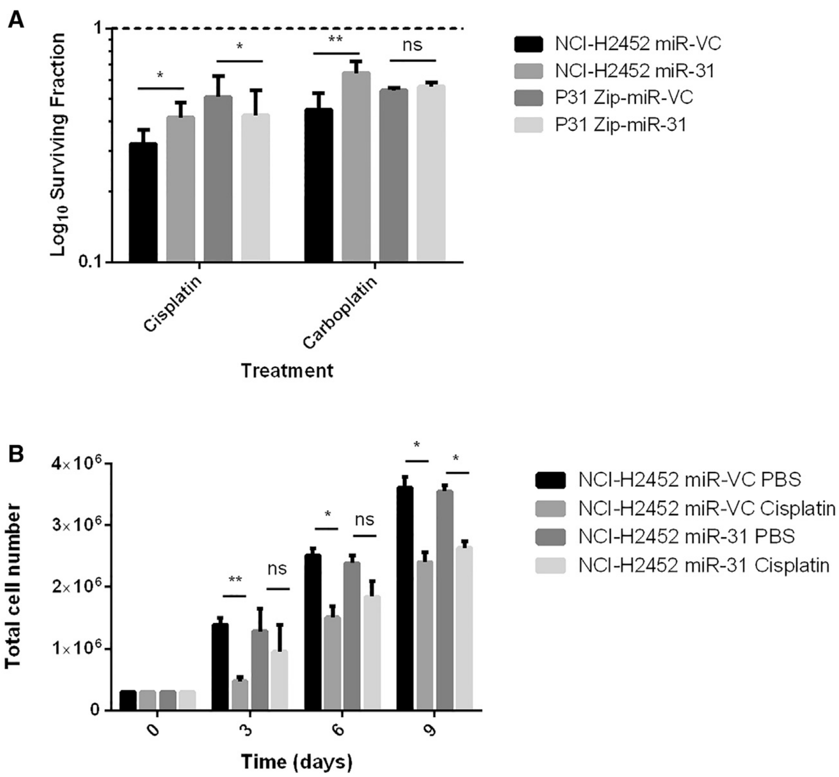

Figure 1. miR-31 Modulation in MPM Cells Alters Cellular Sensitivity in Response to Platinum-Based Chemotherapy Treatment

(A) Clonogenic analysis of miR-31 reintroduction illustrated a significant difference ($p = 0.0028$) between the surviving fractions of miR-VC-transfected cells treated with 1 μM cisplatin (IC_{50} dose) for 24 hr and the miR-31-transfected equivalent ($n = 7$). Furthermore, there is a significant difference ($p = 0.0084$) between the surviving fractions of P31 Zip-miR-VC-transfected cells treated with 2 μM cisplatin (IC_{50} dose) and the Zip-miR-31 equivalent ($n = 3$). miR-31 reintroduction also significantly increases the surviving fraction of NCI-H2452 cells treated with carboplatin ($p = 0.0073$), and suppression of miR-31 significantly increases sensitivity to carboplatin using IC_{50} doses ($p = 0.0198$) ($n = 3$). The dashed line represents cells treated with vehicle control (PBS). (B) Assaying cumulative proliferation with cisplatin treatment revealed a significant decrease in proliferation at all time points in miR-VC cells, whereas miR-31 cells appear less sensitive to the chemotherapeutic agent. The reintroduction of miR-31 in NCI-H2452 cells alters cellular proliferation in response to cisplatin treatment. NCI-H2452 miR-VC cells treated with 1 μM cisplatin have a significant difference in proliferation compared to NCI-H2452 miR-VC untreated cells at day 3 ($p = 0.0027$), day 6 ($p = 0.0366$), and day 9 ($p = 0.0191$) ($n = 3$). There is no significant difference (ns) between the NCI-H2452 miR-31 cells treated with 1 μM cisplatin until day 9 ($p = 0.0306$) and the untreated equivalent ($n = 3$). Data are presented as the mean ± SEM. $^{*}p < 0.05$; $^{**}p < 0.01$.

With the observed reduction in DNA damage and enhancement of cellular resistance to chemotherapy, miR-31 altered uptake or efflux of chemotherapeutics was investigated. Surprisingly, the intracellular level of platinum was increased in NCI-H2452 cells reconstituted with miR-31 (Figure 3). Studies have implicated the importance of influx and efflux of drugs in chemoresistance,[29,30] which directed us to investigate cisplatin flux. No statistically significant differences in the expression of the efflux proteins ATP7A and ATP7B were ascertained (Figure S8); however, there appeared to be a trend ($p = 0.0722$) toward increased expression of CTR1 (Figure 4), a copper transporter known to facilitate influx of cisplatin into the cell.[31] This increase in influx could explain, at least partly, the increased overall concentration of platinum in the miR-31-expressing cells, yet this did not explain the observed resistant phenotype and attenuated DNA damage. Previously, oxidant and antioxidant levels have been linked to resistance to treatment and detoxification of platinum-based therapies.[32,33] To determine whether oxidant and antioxidant levels contributed to resistance, reactive oxygen species (ROS) generation and glutathione levels were assessed. There were no significant changes between vector control and miR-31-expressing cells (Figure S7), indicating that miR-31-enhanced chemoresistance is largely independent of ROS biology.

miR-31 Modulates Nuclear Accumulation of Cisplatin

To further attempt to determine how miR-31-expressing MPM cells remained resistant despite an increase in the level of intracellular cisplatin, subcellular fractionation was employed to separate the organelles of the cells and determine the platinum burden in each fraction. The nuclear fraction was collected and analyzed via inductively coupled plasma mass spectrometry (ICP-MS) to determine whether there was a difference in accumulation of platinum in the nuclear region, where it would be expected to promote cross-linking damage. There was a decrease by approximately 50% in nuclear accumulation of cisplatin observed upon miR-31 re-expression in NCI-H2452 cells (Figure 5B). In addition, the lysosomal fraction illustrated a 0.28 ± 0.07 ppm increase in platinum concentration (Figure 5A). The alterations in γH2A.X supported this finding, collectively demonstrating that miR-31 regulates nuclear transport, whereby trafficking of cisplatin to the nucleus is reduced. This limits damage to DNA indicative of chemotherapeutic treatment, ultimately conferring a survival advantage.

To explain the observed more resistant phenotype, despite a greater concentration of platinum within the cellular environment and less accumulation within the nuclear region, the literature was thoroughly reviewed. There are many routes by which accumulation within the cellular environment may be facilitated, including the efficiency of exosome packaging of cisplatin,[34] changes in the structure of the nuclear region,[35] rearrangements of cytoskeletal components,[36] and lysosomal transport.[24] Established links have been found among miR-31 expression, resistance to therapy, and the lysosomally bound drug transporter ABCB9.[37,38]

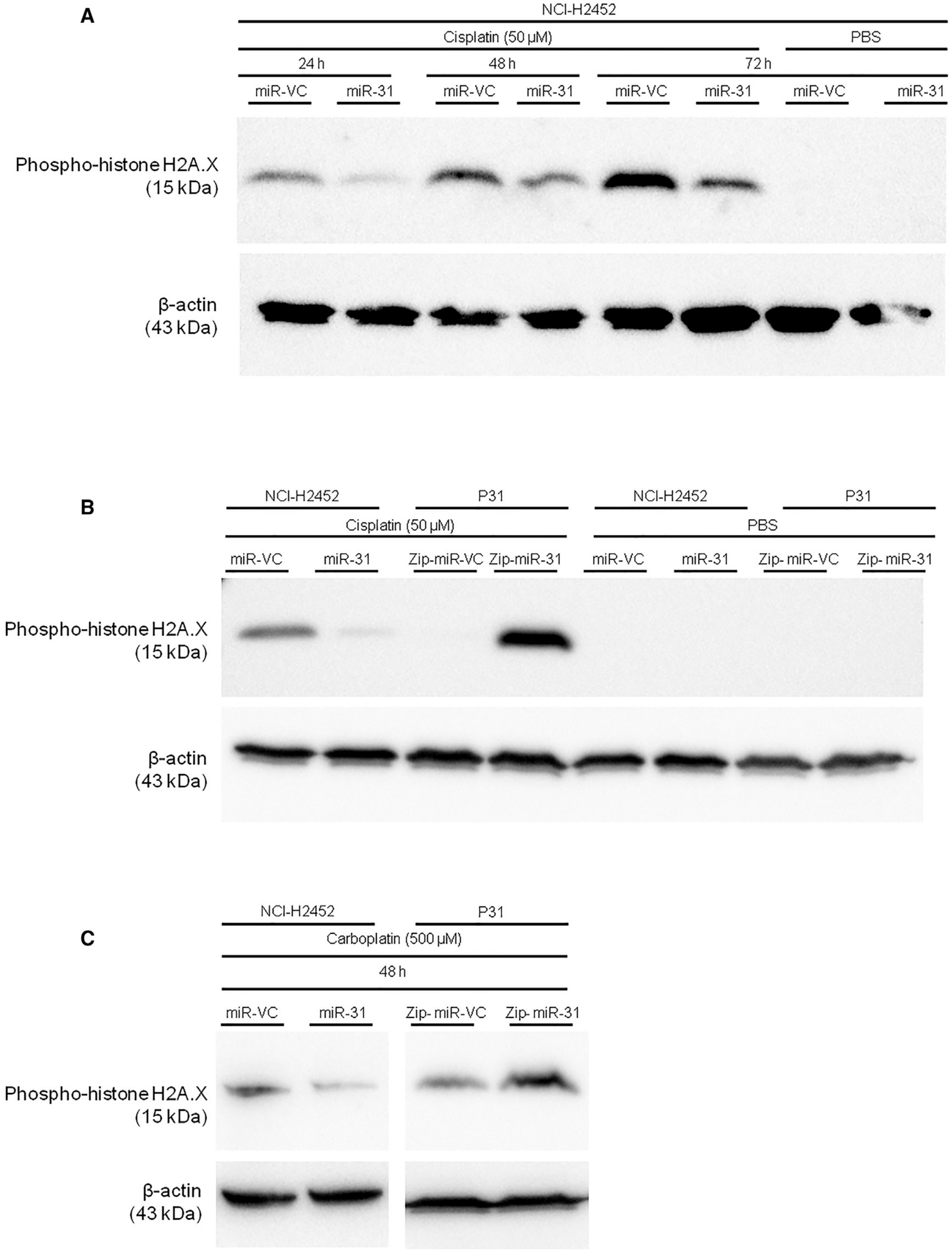

Figure 2. The Expression of miR-31 Correlates with the Amount of DNA Damage Incurred when Treated with Platinum-Based Chemotherapeutics
(A) Representative western blot time course for γH2A.X as a marker of DNA damage with cisplatin treatment (50 μM). Across all time points, it is evident that levels of γH2A.X decreased in miR-31-positive cells. (B) Representative western blot for γH2A.X with cisplatin treatment (50 μM). The confirmation of a reduction in DNA damage with miR-31 can be viewed in the second band (left to right), with an increase in DNA damage evident in the miR-31-suppressed P31 cell line. (C) Representative western blot for γH2A.X with carboplatin treatment (500 μM).

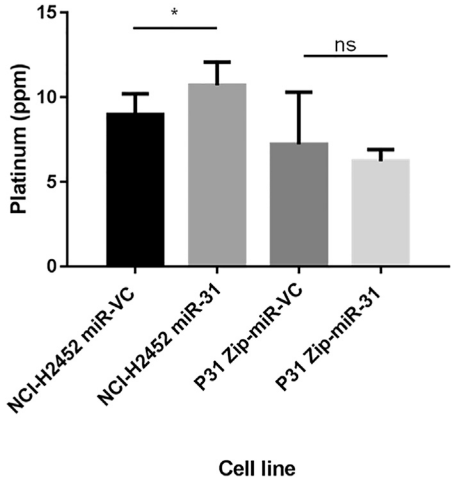

Figure 3. Intracellular Cisplatin Content Is Increased with miR-31 Reintroduction

ICP-MS analysis of NCI-H2452 and P31-transfected cell lines treated with 50 μM cisplatin for 24 hr. There is a significant ($p = 0.0112$) increase in the levels of platinum in NCI-H2452 miR-31 cells compared to miR-VC equivalent, but there is no change in overall platinum levels with miR-31 suppression ($n = 3$). Data are presented as the mean ± SEM. *$p < 0.05$; **$p < 0.01$; ns, not significant.

Lysosomally Bound ABCB9 Is Upregulated with miR-31 Re-expression in MPM Cells, Potentially via an OCT1-Mediated Mechanism in the Extranuclear Compartment

With an increase in overall concentration of cisplatin, increased drug influx, and reduced nuclear accumulation of the drug, the potential capability of miR-31-expressing cells to sequester cisplatin into cytosolic organelles within the cell was investigated. One route by which cells can sequester cytotoxic drugs from the nucleus is through packaging into intracellular vesicles such as lysosomes.[23] An association between miR-31 and the lysosomally bound transporter ABCB9 had been previously established in non-small cell lung cancer (NSCLC),[38] thus prompting an investigation as to whether miR-31 may modulate ABCB9 expression in MPM cells, which consequently may regulate cisplatin transport across the lysosomal membrane. Here, it was identified that there was an upregulation of ABCB9 at both the gene (Figure 6A) and the protein (Figures 6B and 6C) level upon reintroduction of miR-31. This was supported by immunofluorescent studies examining intracellular ABCB9 localization (Figures 6D and 6E). To establish whether the change in ABCB9 expression was accounted for by an increase in the overall burden of lysosomes within the miR-31-expressing cells, the expression of the lysosomal marker LAMP1 was analyzed. It was established that there were no changes in LAMP1 expression, as determined by immunofluorescence and western blot, supporting a specific upregulation of the ABCB9 transporter rather than an increase in the density of lysosomes in the NCI-H2452 miR-31-expressing cell population (Figures 6B–6D and 6F).

Because the mechanism of action for miRNAs typically involves the negative regulation of target genes at the post-transcriptional level, the observed upregulation of ABCB9 upon miR-31 reintroduction indicated the activity of a potential intermediate negative regulator of ABCB9 expression. Here, bioinformatic tools were used to identify potential negative transcriptional regulators of ABCB9, which may be altered by miR-31 (Figure S10; Table S1). OCT1 has previously been noted as a bipotential transcription factor,[22] which we propose, within our system, is a negative regulator of *abcb9* transcription. In line with the present model, it was observed that there was a concomitant reduction in OCT1 expression upon miR-31 reintroduction into NCI-H2452 cells (Figure 7). This may connote miR-31 targeting OCT1, facilitating the increased transcription of downstream proteins such as CTR1 and ABCB9, both of which have binding sites for OCT1.

Direct ABCB9 Overexpression Promotes Chemosensitivity of MPM Cells, Independent of miR-31 Expression

With a correlation between miR-31 reintroduction and ABCB9 overexpression possibly modulating the intracellular accumulation of platinum-based therapy, independent overexpression of ABCB9 was performed in the miR-31 null NCI-H2452 cell line (Figure S11). Surprisingly, overexpression of ABCB9 led to greater levels of γH2A.X induction, suggesting that the high ABCB9-expressing clones (C2 and C3) may be more chemosensitive (Figure 8A). Supporting this, when the clonogenic capacity of the ABCB9-overexpressing NCI-H2452 cells was assessed with a clinically relevant dose of cisplatin, a significantly more chemosensitive phenotype was displayed. The NCI-H2452 ABCB9 overexpressing population had a surviving fraction of 64 ± 19, compared to 93 ± 9 in the vector control (termed EX-NEG) population, meaning ABCB9-overexpressing cells were on average 29% more sensitive than the vector control equivalent (Figure 8B).

In addition, as a measure of cisplatin-induced DNA damage, γH2A.X induction was significantly reduced in NCI-H2452 cells with co-overexpression of ABCB9 and miR-31 (Figure 8A). This indicates that ABCB9 overexpression in the presence of miR-31 is not sufficient to confer a chemosensitive phenotype, thereby inferring that while miR-31 expression promotes ABCB9 expression, the miR-31-mediated chemoresistant phenotype is independent of ABCB9.

DISCUSSION

It is apparent that the role of miR-31 within differing tumor types is multi-faceted and its exact functions remain unclear, with context-dependent evidence supporting both oncogenic and tumor-suppressive functions.[18,39,40] Within MPM, deletion of the fragile site at which miR-31 is encoded has been correlated with poor prognosis;[17] however, it has also been reported that miR-31 expression is allied with aggressive tumor subtypes in patient cohorts.[19] Surprisingly, the present study has determined that miR-31 expression in MPM promotes resistance to platinum-based therapy in vitro. However, the suppression of miR-31 in the P31 cell line led to no significant difference in the surviving fraction when treated with carboplatin. Carboplatin relies upon passive diffusion to enter the cell;[41] hence, potential pleiotropic alterations due to the suppression of miR-31 may modulate the ability of the molecule to enter the cellular environment.

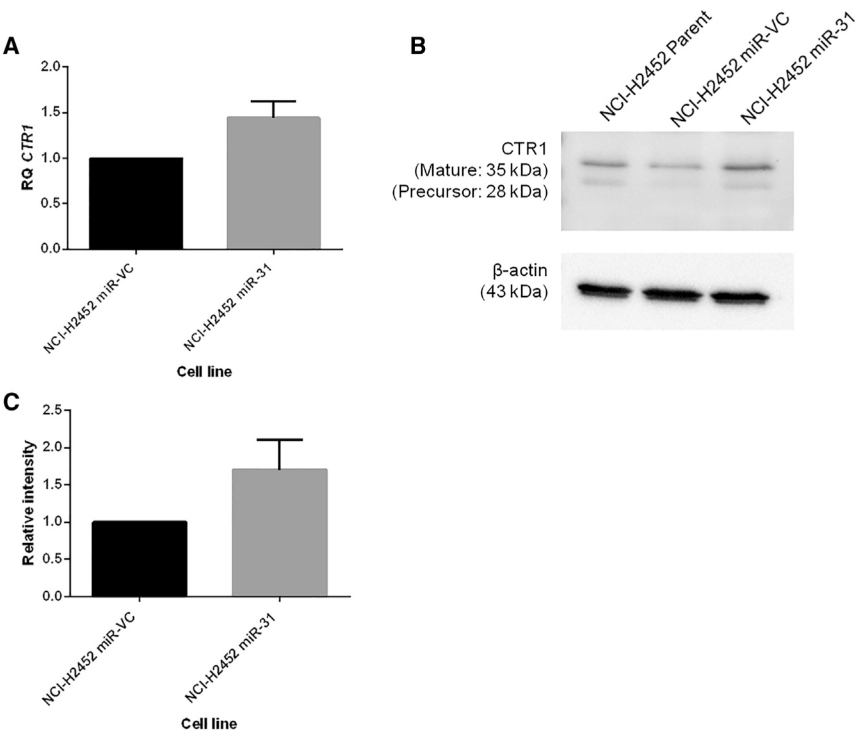

Figure 4. miR-31 Rehabilitation in MPM Cells May Alter the Expression of Drug Influx Transporter CTR1

(A) Expression levels of drug influx transporter *slc31a1* (CTR1) were analyzed via qPCR. Relative quantity (RQ) relates to relative fold change (n = 4). (B) Representative western blot illustrating CTR1 expression to be moderately amplified by miR-31 reintroduction. (C) Densitometry analysis revealing upregulation of the CTR1 protein (n = 3). Data are presented as the mean ± SEM.

Data from this study indicate the loss of miR-31 in MPM tumors may confer a chemosensitive phenotype. Although the data represented here are in in vitro-based 2D models, there may be allied results in vivo. In contrast to the initial hypothesis, the data support the alternative hypothesis that miR-31 loss in MPM confers a positive prognostic influence. The potential mechanism by which miR-31 appears to mediate resistance relies upon regulation of intracellular transport. Dependence upon nuclear transport has previously been noted in breast cancer,[42] with associations between altered transport of platinum-containing agents within the cellular environment and resistance to therapy being comprehensively reviewed.[43] Laurila et al.[44] detailed the involvement of a nuclear transport protein, KPNA7, with promotion of malignancy in pancreatic cancer. The silencing of KPNA7 led to the inhibition of malignancy in pancreatic cell lines, which highlights the importance of transportation from the cytoplasmic to the nuclear compartment and its involvement with resistance to therapy.

Cellular accumulation of chemotherapeutics has been comprehensively reviewed.[45] With a higher overall amount of platinum in miR-31-expressing cells (Figure 3) and a reduction in the concentration of platinum in the nuclear fraction (Figure 5), the question remained as to how cells were able to survive an increased intracellular concentration of cisplatin. There is evidence supporting that miRNAs can mediate cellular sequestration through the alteration of calcium signaling or the mediation of multi-drug-resistant proteins.[46–48] Here, following further fractionation of cellular components, a change in lysosomal accumulation was observed; this promoted the investigation of possible drug transporters that were bound to lysosomes and promoted the investigation as to whether miR-31-expressing cells had a higher aggregate burden of lysosomes. Pennati et al.[47] showed that miR-205 replacement in prostate cancer cells downregulated lysosome function and protein trafficking, leading to alterations in the autophagic flux of cells, which changed the detoxifying capabilities by which cells become cisplatin resistant. Drayton et al.[49] correlated reduced miR-27a expression with the cysteine and glutamate exchanger SLC7A11. Cisplatin-resistant bladder cancer cell lines were resensitized by initiating miR-27a expression, or reducing the activity of SLC7A11 via siRNA, which supports the findings that miRNAs can regulate cellular transporters, thus connoting regulation of cellular chemoresistance.

The lysosomally bound drug transporter ABCB9[37] has been identified as a modulator of resistance, with up- or downregulation of the protein enhancing or reducing response to therapeutics.[50] Surprisingly, while ABCB9 appears to be increased with the miR-31 overexpressing more resistant phenotype, upon manipulation of ABCB9 in miR-31 null NCI-H2452 cells, there is a sensitizing effect to cisplatin treatment. Dong et al.[38] examined the relationship between miR-31 and ABCB9. The investigation established a link in NSCLC cisplatin resistant cell lines having higher expression of miR-31, with a downregulation of the drug transporter ABCB9, potentiating that miR-31 directly targeted ABCB9 and so repressed its translation. However, there is an elevation in ABCB9 protein associated with the miR-31-mediated chemoresistant phenotype. Contextually, this may be explained by the pleiotropic function of microRNAs, including miR-31 targeting the mRNA of a transcription factor that regulates ABCB9. In short, the increase of miR-31 expression may lead to a downregulation of a potential negative regulator of transcription, effectively taking off a transcriptional brake, which may lead to an increase in the expression of target proteins such as ABCB9. Here, we potentially identified OCT1 as a candidate for such a theory. Dong et al.[38] also focused upon the inhibition of ABCB9 contributing to cisplatin resistance in NSCLC, and our reciprocal ABCB9-overexpression data in MPM supports these previous findings.

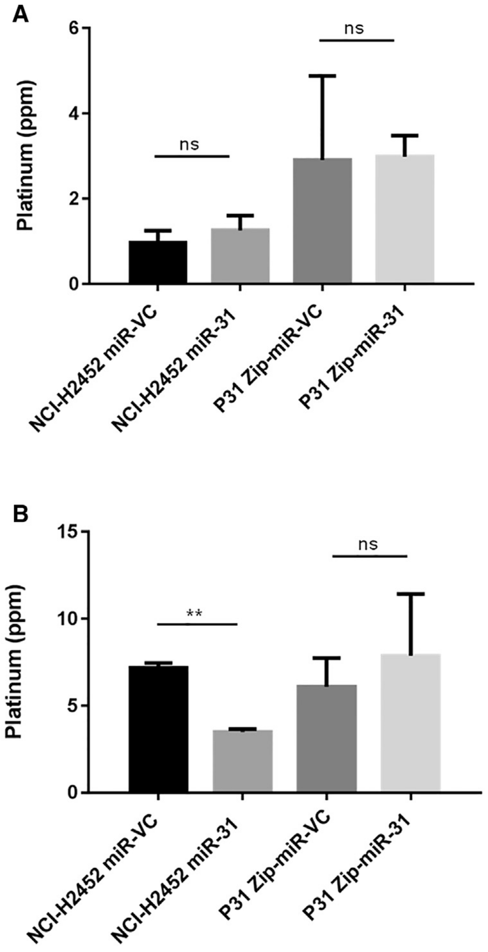

Figure 5. miR-31 Status Affects the Cisplatin Content of Specific Organelles within the Intracellular Environment

(A) Isolation of the lysosomal fraction following 50 μM cisplatin treatment for 24 hr illustrated a trend toward increased cisplatin concentration with miR-31 re-introduction; however, this is not significant (ns = not significant) (n = 3). (B) ICP-MS analysis indicated a significant difference (p = 0.0028) in platinum concentration within the nuclear fraction of miR-31-expressing cells following 50 μM cisplatin treatment for 24 hr. A trend toward an increased concentration within the miR-31-suppressed population is evident (n = 3). Data are presented as the mean ± SEM. *p < 0.05; **p < 0.01.

We have shown miR-31 mediation of a chemoresistant phenotype within in vitro studies of MPM, with an observed increase in ABCB9 expression. However, when ABCB9 is overexpressed independently of miR-31, an increase in sensitivity is observed, opposing the initial hypothesis and perhaps indicating that the increase in ABCB9 is passive, because miR-31 is mediating other molecules that potentially contribute more to the regulation of cisplatin intracellular accumulation. This implies that ABCB9 expression is important within the context of MPM; however, ABCB9 upregulation, in this case, does not contribute to the specific pathway mediated by miR-31 to enhance MPM chemoresistance. Although the alteration in sensitivity to cisplatin with ABCB9 overexpression was apparent, there can be limitations to the significance of this observation when the analysis of the model used is completed. As in Figure S12, the miR-31-driven and miR-31-independent overexpression of ABCB9 led to differing localization of the transporter within the cellular environment, which may explain the differing response observed.

The movement of platinum-based chemotherapeutics within the intracellular environment is widely characterized, with CTR1, ATP7A, and ATP7B known to play significant trafficking roles.[51,52] Although no significant changes are apparent for the influx and efflux transporters CTR1 (Figure 3), ATP7A, and ATP7B (Figure S8), there may be contributions to the overall phenotypic resistance observed with miR-31 reintroduction. Stordal et al.[53] and Kalayda et al.[54] illustrated the importance of localization of these key proteins, which can modulate accumulation and orchestrate the sequestration of cisplatin within resistant cells, although there is typically no change in overall expression. ATP7A and ATP7B have been established as Golgi network transporters; however, within resistant models, a translocation to outer vesicular structures has been noted,[54] signifying that while the present results show no gross difference, these molecules may be modulating sensitivity through alterations in their localization.

The effect of miR-31 reintroduction on increasing chemoresistance was in agreement with our initial hypothesis; however, the group had previously associated miR-31 overexpression with increased sensitivity to therapy in esophageal adenocarcinoma.[18] Although unanticipated, a potentially novel mechanism behind enhanced resistance, which may potentiate a modified strategy of treatment in the future, has been uncovered. Many MPM patients are inherently resistant to chemotherapy, and most have extremely poor prognosis; this has driven the field to find an alternative therapeutic or enhance the ability of the readily available therapeutics to combat this disease. Prospectively, the consequence of further investigating this mechanism within an in vivo system may lead to the ability to screen patients for miR-31 status. Patients who express high levels of miR-31 could be stratified to have an antagomir administered to suppress miR-31 expression, which could mean the efficiency of platinum-based chemotherapy cytotoxicity would be enhanced. Zhang et al.[55] co-treated with both paclitaxel and antagomir miR-10b in breast cancer cell lines, using the chemotherapeutic to treat the primary tumor and suppressing miR-10b to decrease metastasis. The results were promising, and they concluded administration was successful for both the antagomir and the paclitaxel via a liposomal-based system.

miRNA treatment in the form of nucleic acid-modified DNA phosphorothioate antisense oligonucleotides has already entered human clinical trials in treatment of disease, although not in cancer. miR-122, the abundant liver-expressed miRNA, is sequestered by the oligonucleotide and bound in a duplex, which inhibits endogenous function within hepatitis C virus infection. Results thus far have shown promise, with long-standing dose-dependent decreases in infection levels without evidence of acquired resistance.[56] In relation to mesothelioma, there has been progress in clinical trials using

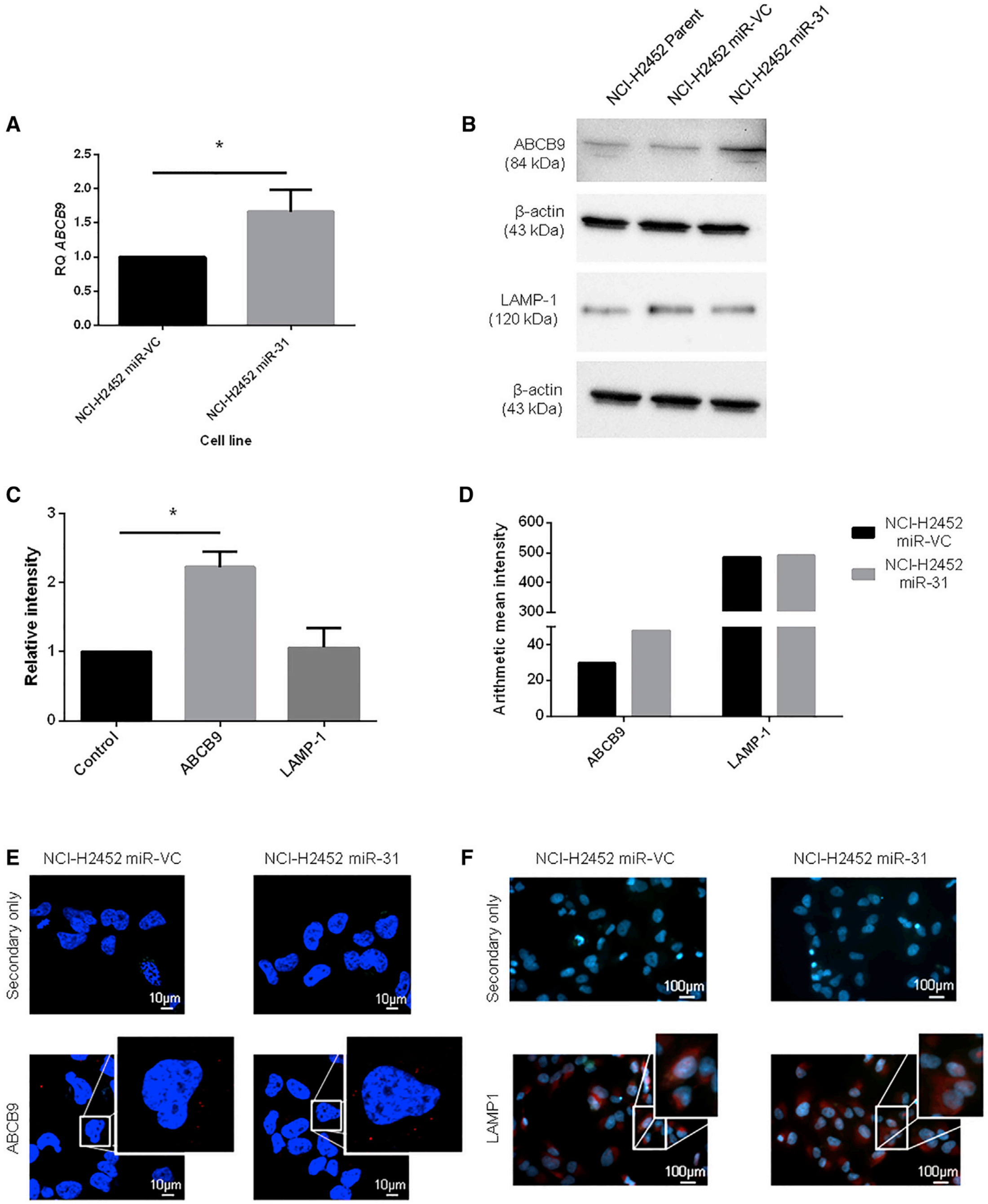

Figure 6. Reintroduction of miR-31 Affects Lysosomal Drug Transport

(A) Expression levels of the drug influx transporter *abcb9* were analyzed via qPCR. There is a significantly greater relative expression level (p = 0.0251) of ABCB9 in miR-31-transfected cells compared to the miR-VC-transfected equivalent (n = 4). RQ relates to relative fold change. (B) Representative western blot illustrating an increase in ABCB9 expression level with miR-31 re-expression, with no apparent change in the lysosomal marker LAMP1. (C) Densitometry analysis revealing significant (p = 0.0325) upregulation of ABCB9 (n = 3). (D) Mean signal intensities of NCI-H2452-transfected cells immunofluorescently stained with ABCB9 or LAMP1, with intensities captured in the Texas red channel for the images in (E). (E and F) Immunofluorescent images showing slight alteration in ABCB9 (E) or LAMP1 (F) staining (red); nuclei are stained with DAPI (blue). Images were captured on LSM 710 with a 63×/1.40 oil DIC M27 objective for ABCB9 images and a 10×/0.25 Ph1 objective for LAMP1 images. Data are presented as the mean ± SEM. *p < 0.05; **p < 0.01.

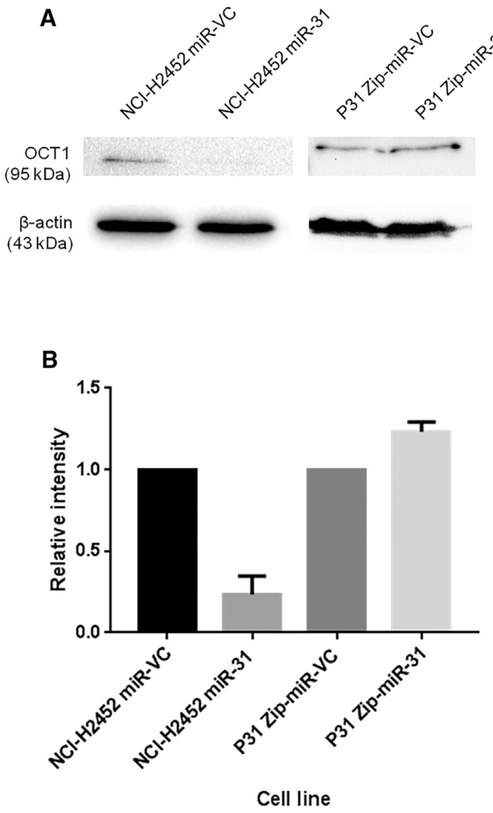

Figure 7. The Bipotential Transcriptional Regulator, OCT1, Can Be Associated with miR-31 Expression

(A) Representative western blot illustrating the downregulation of a potential negative regulator of both CTR1 and ABCB9 expression, OCT1, with miR-31 reintroduction. Suppression of miR-31 does not affect expression of OCT1. (B) Densitometry analysis of OCT1 expression with miR-31 reintroduction (NCI-H2452) or miR-31 suppression (P31) (n = 2). The data demonstrate a large decrease in OCT1 with miR-31 re-expression, potentiating miR-31-mediated downregulation of OCT1 and leading to upregulation of CTR1 and ABCB9 expression. Data are presented as the mean ± SEM.

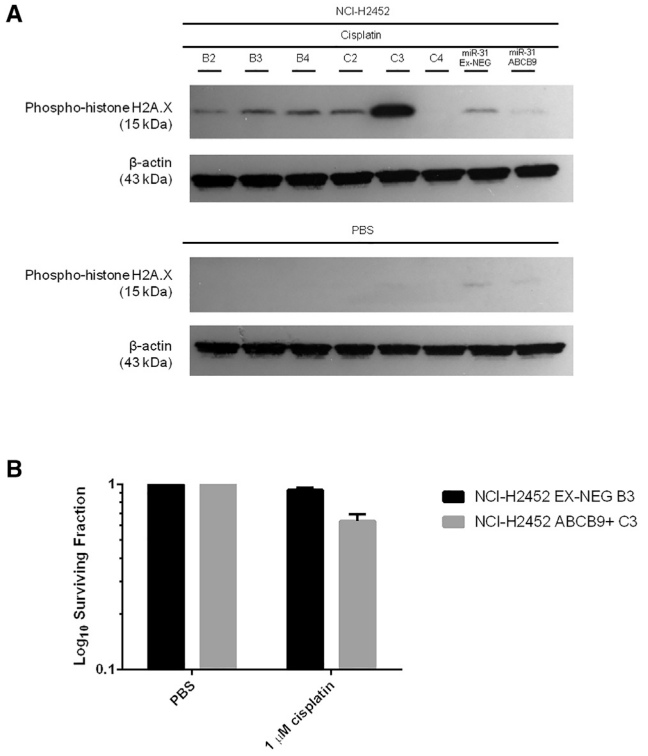

Figure 8. Overexpression of the Lysosomal Drug Transporter ABCB9 Affects Chemosensitivity, Independent of miR-31

(A) Representative western blot demonstrating the increased levels of γH2A.X with high expression of ABCB9 in the parent NCI-H2452 cell line (the ABCB9 overexpression cell line is termed NCI-H2452 ABCB9+). Clones B2–B4 correlate to EX-NEG vector control clones (vector control for ABCB9 overexpression model is termed NCI-H2452 EX-NEG). Clones C2 and C3 correlate to high expressers of ABCB9, and C4 is a low expresser of ABCB9. All clones were treated for 24 hr with 50 μM cisplatin. (B) ABCB9 overexpression sensitizes miR-31 null NCI-H2452 cells to cisplatin after treatment with 1 μM cisplatin for 24 hr, with near significance reached (p = 0.0516) (n = 3). Data are presented as the mean ± SEM.

miRNAs to modulate the disease, Kao et al.[57] reported both a metabolic and radiological response with miR-16-based mimics. The further development of TargomiRs, miRNAs delivered via bacterial minicells, to treat thoracic cancers has also shown promise.[58]

Within the context of this study, miR-31 expression in MPM facilitates resistance to platinum-based chemotherapy. miR-31-mediated changes in ABCB9 expression and lysosomal uptake of cisplatin are not sufficient to promote chemoresistance, indicating that miR-31 mediates chemoresistance in MPM through a yet-unidentified molecular mechanism involving reduced nuclear trafficking of chemotherapeutic agents; however, the vector-driven overexpression may limit the significance of the ABCB9 arm in this investigation. Prospectively, although not within the remit of the current investigation, this research would benefit from support within the in vivo setting with the use of an MPM mouse model. In addition, data would be supported with MPM patient-derived samples with and without chemotherapeutic treatment to analyze miR-31 status, and potentially OCT1 or ABCB9 expression; these aspects would strengthen the impact of the current research. Our data suggest that while deletions in chromosome 9p21.3 may be associated with an overall poor prognosis, the specific loss of miR-31 from this region may not contribute to the chemoresistance observed in MPM patients. Screening patients for miR-31 expression status and corresponding suppression of the miRNA may promote enhanced sensitivity to platinum-based chemotherapeutics, improving patient outcomes.

MATERIALS AND METHODS

Cell Culture

The MPM epithelioid subtype cell lines NCI-H2452 and P31 were gifts from Dr. Steven Gray (Department of Oncology and Clinical Medicine, Trinity Translational Medicine Institute, Trinity Sciences Health Centre, St. James's Hospital). Cell lines were maintained in RPMI 1640 medium (Lonza) supplemented with 10% fetal bovine serum (HyClone), 1% penicillin/streptomycin (Lonza), and 1%

GlutaMAX (Gibco). Cells were maintained in humidified incubators at 37°C, 5% CO_2. Regular mycoplasma testing was carried out using the MycoAlert Mycoplasma Detection Kit (Lonza), with no contamination evident.

Cytotoxic Reagents

Cisplatin was purchased from Acros Organics (Thermo Fisher Scientific) and solubilized in sterile PBS (Lonza). Carboplatin was purchased from Selleckchem (Stratech) and solubilized in sterile PBS (Lonza). Aliquots were stored at −20°C and thawed immediately before use.

miRNA Transfection

Transfections were performed using Lipofectamine 2000 reagent from Invitrogen according to the manufacturer's instructions. miR-31 overexpression plasmid (MI0000089) and vector control (PCMVMIR) were purchased from Origene and stably transfected into the NCI-H2452 cell line under 500 μg/mL G418 (Thermo Fisher Scientific) selection for ∼21 days. Zip-miR-31 plasmid (MZIP31-PA-1) and Zip vector control (MZIP000-PA-1) were purchased from SBI and stably transfected into the P31 cell line under 3 μg/mL puromycin (Sigma Aldrich) selection for 10 days.

ABCB9 Transfection

Lipofectamine 2000 reagent from Invitrogen was used according to the manufacturer's instructions. ABCB9 (EX-T8156-M68) overexpression plasmid and vector control (EX-NEG-M68) were purchased from GeneCopoeia and stably transfected into the NCI-H2452 cell line under 2 μg/mL puromycin (Sigma Aldrich) selection for 14 days. The surviving cells were kept under 0.5 μg/mL puromycin maintenance selection while culturing.

Clonogenic Assay

Clonal survival was determined by seeding NCI-H2452 (5×10^2–1.5×10^3) or P31-transfected cells (5×10^2–1×10^3) into 6-well plates and allowing them to adhere overnight. Cells were subjected to cisplatin treatment using established doses (Figure S3) for 24 hr, and then treatment was removed and complete RPMI was applied. Plates were incubated for 8–10 days after seeding. Colonies were fixed and stained with crystal violet solution (0.1% w/v crystal violet, 70% v/v methanol, 30% v/v deionized water), and wells were washed with water until colonies were distinct. Colonies were counted using the Oxford Optronix GelCount instrument and optimized compact hough and radial map (CHARM) image processing algorithms for each cell line.

Plating efficiency was calculated as the colony count divided by the number of cells seeded. Surviving fraction was therefore calculated as the colony count divided by the plating efficiency of the control and multiplied by the number of cells seeded.

Cumulative Proliferation Assay

A proliferation assay was employed wherein 3×10^5 cells were seeded into 10 cm^2 tissue culture dishes and allowed to adhere overnight. Cisplatin treatment was then applied for 24 hr. Cells were reseeded at 3×10^5 every 3 days, for a total of 9 days, and a cumulative cell count was taken.

ICP-MS

Cells were treated with 50 μM cisplatin for 24 hr, after which cells were harvested and counted. Cells (1×10^6) were incubated in HNO_3 for 72 hr at room temperature, and following incubation, HCl was added to each sample to form aqua regia at a ratio of 1:3, ensuring total platinum was in solution. Samples were analyzed by Perkin Elmer DRCII ICP-MS. Standard curves were generated using aqueous serial dilutions of known standards. Each measurement taken was representative of three technical replicates from an individual sample.

Subcellular Fractionation

Cells were subjected to homogenization using a sucrose-based solution (250 mM sucrose, 50 mM Tris-HCl, 5 mM $MgCl_2$). Disruption of the cellular membrane was completed with five strokes of a Dounce homogenizer. Homogenized cells were then subjected to 600 × *g* for 3 min to isolate nuclei; further fractionation at 6,000 × *g* for 8 min separated mitochondria, lysosomes, and peroxisomes.

Reverse Transcription and qPCR

Total RNA was isolated from cells using the RNeasy Mini kit (QIAGEN) according to the manufacturers' instructions. Quantification of total RNA, quality, and content was determined by Nanodrop Lite (Thermo Fisher Scientific). RNA was reverse transcribed using the QuantiTect Reverse Transcription kit (QIAGEN) according to the manufacturers' instructions. QuantiTect and the miScript SYBR Green PCR Master mix (QIAGEN) were used to assess mRNA and miRNA levels using real-time qPCR (Applied Biosystems). Commercially available QuantiTect (CTR1, QT00099267; ABCB9, QT00089047; ATP7A, QT00075852; and ATP7B, QT00075782) and miScript (miR-31, MS00003290) primer assays were employed for both the genes of interest and the endogenous controls (B2M, QT00088935, for mRNA and RNU6, MS00033740, for miRNA).

Western Blot

Cells were lysed in cold radioimmunoprecipitation assay (RIPA) buffer (50 mM Tris-HCl [pH 8], 1% Triton X, 0.5% sodium deoxycholate, 0.1% SDS, 150 mM NaCl) with the addition of protease and phosphatase inhibitor tablets (Pierce). Protein concentration was quantified using the BCA protein assay kit (Pierce), with 50 μg of protein per sample loaded onto gels. Proteins were separated on 7.5% or 10% SDS-PAGE gels, transferred onto polyvinylidene fluoride (PVDF) membrane (Thermo Fisher Scientific), and probed for CTR1 (sc-66847) used at 1:1,000, ABCB9 (sc-393412) used at 1:1,000, LAMP1 (sc-17768) used at 1:1,000, OCT1 (sc-293181) used at 1:1,000, β-actin (sc-130300) used at 1:10,000 (Santa Cruz Biotechnology), and γH2A.X (9718) used at 1:1,000 (Cell Signal), followed by incubation with anti-mouse horseradish peroxidase (HRP)-conjugated secondary antibody (P0260) used at 1:2,000 (Dako) or anti-rabbit HRP-conjugated secondary antibody (sc-2004) used at 1:2,000

(Santa Cruz Biotechnology). Bands were detected using Clarity western enhanced chemiluminescence (ECL) substrate (Bio-Rad) and visualized using the ChemiDoc imaging system (Molecular Imager ChemiDoc XRS with Image Lab 3.0). Densitometric analysis was performed with Image Lab 3.0 software (Bio-Rad).

Nuclear Extraction

Cells were harvested and washed with ice-cold NEB buffer A (10 mM HEPES [pH 7.9], 1.5 mM $MgCl_2$, 10 mM KCl, 1 mM DTT, protease inhibitor cocktail (PIC), ultrapure H_2O) and pelleted at 20,000 $\times$ g for 2 min. Cells were washed a further two times with NEB buffer A. Cell lysis was achieved by resuspending cells in NEB buffer A plus 0.1% (v/v) NP40 with incubation on ice. Lysate was separated by centrifugation at 20,000 $\times$ g for 15 min, followed by nuclear lysis with NEB buffer C (20 mM HEPES [pH 7.9], 1.5 mM $MgCl_2$, 420 mM NaCl, 1 mM DTT, PIC, ultrapure H_2O). Insoluble material was removed via centrifugation at 20,000 $\times$ g for 15 min.

Immunofluorescence

NCI-H2452 miR-VC and NCI-H2452 miR-31 cell lines were seeded on glass coverslips within a 6-well plate at a density of 4×10^5 per well. Cells were fixed with 4% formaldehyde and blocked with blocking solution (1× PBS, 5% serum, 0.3% Triton X-100). Primary antibody was applied in antibody dilution buffer (1× PBS, 1% BSA, 0.3% Triton X-100) and incubated at 4°C overnight. Following washing with 1× PBS, cells were incubated with Alexa Fluor 555 (Thermo Fisher Scientific) secondary antibody diluted in antibody dilution buffer for 2 hr at room temperature. Coverslips were reverse mounted onto glass slides with ProLong Gold Antifade Mountant with DAPI (Thermo Fisher Scientific). Slides were left to cure overnight before imaging on Zeiss LSM710 AxioObserver Confocal microscope using the Plan Apochromat 63×/1.40 oil differential interference contrast (DIC) M27 objective for ABCB9 images and the LD A-Plan 10×/0.25 Ph1 objective for LAMP1 images. Images were captured with AxioCamIC with a 0.5× camera adapter.

Bioinformatics

Gene promoter bioinformatics analysis was performed using the DECODE database (http://www.sabiosciences.com/chipqpcrsearch.php?app=TFBS). Genes of interest were inserted, with analysis showing the potential binding sites of more than 200 transcription factors 20 kb upstream and 10 kb downstream of the gene.

Statistical Analysis

Data were statistically analyzed using InStat3 software. Unless otherwise stated, data are presented as the mean ± SEM of three or more independent experiments. Paired t test was performed on clonogenic and ICP-MS data. Proliferation data were subject to analysis via ANOVA with Tukey's post hoc test. Densitometry and qPCR data were analyzed for significance using one-sample t test, wherein the hypothetical mean was set to 1.0. Significance was considered to be $p < 0.05$.

SUPPLEMENTAL INFORMATION

Supplemental Information includes twelve figures and one table and can be found with this article online at http://dx.doi.org/10.1016/j.omtn.2017.07.001.

AUTHOR CONTRIBUTIONS

H.L.M. designed and conducted the experiments and wrote the manuscript. M.J.L. provided clinical advice and devised the project. S.G.M. devised the project, designed the experiments, edited the manuscript, and provided financial support.

ACKNOWLEDGMENTS

The authors thank Dr. Steven Gray (Department of Oncology and Clinical Medicine, Trinity Translational Medicine Institute, Trinity Sciences Health Centre, St James's Hospital, Dublin, Ireland) for the gift of MPM cell lines, Bob Knight (Department of Chemistry, University of Hull, Hull, UK) for assistance with ICP-MS analysis, and Dr. Jennifer Waby (University of Bradford, Bradford, UK) for aid with confocal microscopy. H.L.M. was supported by a PhD scholarship awarded by the University of Hull and Hull York Medical School.

REFERENCES

1. van Meerbeeck, J.P., Scherpereel, A., Surmont, V.F., and Baas, P. (2011). Malignant pleural mesothelioma: the standard of care and challenges for future management. Crit. Rev. Oncol. Hematol. *78*, 92–111.
2. Weng, C.-H., Ho, P.-Y., Chen, C.-K., and Tsai, W.-K. (2013). Incidentally discovered malignant mesothelioma of the tunica vaginalis. J. Formos. Med. Assoc. *112*, 57–58.
3. Markaki, S., Protopapas, A., Milingos, S., Lazaris, D., Antsaklis, A., and Michalas, S. (2005). Primary malignant mesothelioma of the peritoneum: a clinical and immunohistochemical study. Gynecol. Oncol. *96*, 860–864.
4. Nymark, P., Wikman, H., Hienonen-Kempas, T., and Anttila, S. (2008). Molecular and genetic changes in asbestos-related lung cancer. Cancer Lett. *265*, 1–15.
5. Linton, A., Vardy, J., Clarke, S., and van Zandwijk, N. (2012). The ticking time-bomb of asbestos: its insidious role in the development of malignant mesothelioma. Crit. Rev. Oncol. Hematol. *84*, 200–212.
6. Buikhuisen, W.A., Hiddinga, B.I., Baas, P., and van Meerbeeck, J.P. (2015). Second line therapy in malignant pleural mesothelioma: a systematic review. Lung Cancer *89*, 223–231.
7. Bibby, B.A.S., Reynolds, J.V., and Maher, S.G. (2015). MicroRNA-330-5p as a putative modulator of neoadjuvant chemoradiotherapy sensitivity in oesophageal adenocarcinoma. PLoS ONE *10*, e0134180.
8. Lynam-Lennon, N., Maher, S.G., and Reynolds, J.V. (2009). The roles of microRNA in cancer and apoptosis. Biol. Rev. Camb. Philos. Soc. *84*, 55–71.
9. Maher, S.G., Bibby, B.A.S., Moody, H.L., and Reid, G. (2015). MicroRNAs and cancer. In Epigenetic Cancer Therapy, S.G. Gray, ed. (Academic Press), pp. 67–90.
10. Floor, S.L., Dumont, J.E., Maenhaut, C., and Raspe, E. (2012). Hallmarks of cancer: of all cancer cells, all the time? Trends Mol. Med. *18*, 509–515.
11. Schwarzenbach, H., Nishida, N., Calin, G.A., and Pantel, K. (2014). Clinical relevance of circulating cell-free microRNAs in cancer. Nat. Rev. Clin. Oncol. *11*, 145–156.
12. Fukushige, S., and Horii, A. (2014). Road to early detection of pancreatic cancer: attempts to utilize epigenetic biomarkers. Cancer Lett. *342*, 231–237.
13. De Guire, V., Robitaille, R., Tétreault, N., Guérin, R., Ménard, C., Bambace, N., and Sapieha, P. (2013). Circulating miRNAs as sensitive and specific biomarkers for the diagnosis and monitoring of human diseases: promises and challenges. Clin. Biochem. *46*, 846–860.
14. Chan, E., Prado, D.E., and Weidhaas, J.B. (2011). Cancer microRNAs: from subtype profiling to predictors of response to therapy. Trends Mol. Med. *17*, 235–243.

15. Dacic, S., Kothmaier, H., Land, S., Shuai, Y., Halbwedl, I., Morbini, P., Murer, B., Comin, C., Galateau-Salle, F., Demirag, F., et al. (2008). Prognostic significance of p16/cdkn2a loss in pleural malignant mesotheliomas. Virchows Arch. *453*, 627–635.

16. Ladanyi, M. (2005). Implications of P16/CDKN2A deletion in pleural mesotheliomas. Lung Cancer *49* (*Suppl 1*), S95–S98.

17. Ivanov, S.V., Goparaju, C.M., Lopez, P., Zavadil, J., Toren-Haritan, G., Rosenwald, S., Hoshen, M., Chajut, A., Cohen, D., and Pass, H.I. (2010). Pro-tumorigenic effects of miR-31 loss in mesothelioma. J. Biol. Chem. *285*, 22809–22817.

18. Lynam-Lennon, N., Reynolds, J.V., Marignol, L., Sheils, O.M., Pidgeon, G.P., and Maher, S.G. (2012). MicroRNA-31 modulates tumour sensitivity to radiation in oesophageal adenocarcinoma. J. Mol. Med. (Berl.) *90*, 1449–1458.

19. Matsumoto, S., Nabeshima, K., Hamasaki, M., Shibuta, T., and Umemura, T. (2014). Upregulation of microRNA-31 associates with a poor prognosis of malignant pleural mesothelioma with sarcomatoid component. Med. Oncol. *31*, 303.

20. Bae, H.J., Jung, K.H., Eun, J.W., Shen, Q., Kim, H.S., Park, S.J., Shin, W.C., Yang, H.D., Park, W.S., Lee, J.Y., and Nam, S.W. (2015). MicroRNA-221 governs tumor suppressor HDAC6 to potentiate malignant progression of liver cancer. J. Hepatol. *63*, 408–419.

21. Lu, G., Sun, Y., An, S., Xin, S., Ren, X., Zhang, D., Wu, P., Liao, W., Ding, Y., and Liang, L. (2015). MicroRNA-34a targets FMNL2 and E2F5 and suppresses the progression of colorectal cancer. Exp. Mol. Pathol. *99*, 173–179.

22. Shakya, A., Kang, J., Chumley, J., Williams, M.A., and Tantin, D. (2011). Oct1 is a switchable, bipotential stabilizer of repressed and inducible transcriptional states. J. Biol. Chem. *286*, 450–459.

23. Gorden, B.H., Saha, J., Khammanivong, A., Schwartz, G.K., and Dickerson, E.B. (2014). Lysosomal drug sequestration as a mechanism of drug resistance in vascular sarcoma cells marked by high CSF-1R expression. Vasc. Cell *6*, 20.

24. Gotink, K.J., Broxterman, H.J., Labots, M., de Haas, R.R., Dekker, H., Honeywell, R.J., Rudek, M.A., Beerepoot, L.V., Musters, R.J., Jansen, G., et al. (2011). Lysosomal sequestration of sunitinib: a novel mechanism of drug resistance. Clin. Cancer Res. *17*, 7337–7346.

25. Johansson, D., Andersson, C., Moharer, J., Johansson, A., and Behnam-Motlagh, P. (2010). Cisplatin-induced expression of Gb3 enables verotoxin-1 treatment of cisplatin resistance in malignant pleural mesothelioma cells. Br. J. Cancer *102*, 383–391.

26. Giovannetti, E., Zucali, P.A., Assaraf, Y.G., Leon, L.G., Smid, K., Alecci, C., Giancola, F., Destro, A., Gianoncelli, L., Lorenzi, E., et al. (2011). Preclinical emergence of vandetanib as a potent antitumour agent in mesothelioma: molecular mechanisms underlying its synergistic interaction with pemetrexed and carboplatin. Br. J. Cancer *105*, 1542–1553.

27. Kang, Y., Ding, M., Tian, G., Guo, H., Wan, Y., Yao, Z., Li, B., and Lin, D. (2013). Overexpression of Numb suppresses tumor cell growth and enhances sensitivity to cisplatin in epithelioid malignant pleural mesothelioma. Oncol. Rep. *30*, 313–319.

28. Kuo, L.J., and Yang, L.X. (2008). Gamma-H2AX—a novel biomarker for DNA double-strand breaks. In Vivo *22*, 305–309.

29. Takano, M., Otani, Y., Tanda, M., Kawami, M., Nagai, J., and Yumoto, R. (2009). Paclitaxel-resistance conferred by altered expression of efflux and influx transporters for paclitaxel in the human hepatoma cell line, HepG2. Drug Metab. Pharmacokinet. *24*, 418–427.

30. De Mattia, E., Cecchin, E., and Toffoli, G. (2015). Pharmacogenomics of intrinsic and acquired pharmacoresistance in colorectal cancer: toward targeted personalized therapy. Drug Resist. Updat. *20*, 39–70.

31. Ishida, S., Lee, J., Thiele, D.J., and Herskowitz, I. (2002). Uptake of the anticancer drug cisplatin mediated by the copper transporter Ctr1 in yeast and mammals. Proc. Natl. Acad. Sci. USA *99*, 14298–14302.

32. Bose Girigoswami, K., Bhaumik, G., and Ghosh, R. (2005). Induced resistance in cells exposed to repeated low doses of H2O2 involves enhanced activity of antioxidant enzymes. Cell Biol. Int. *29*, 761–767.

33. Fong, C.W. (2016). Platinum anti-cancer drugs: free radical mechanism of Pt-DNA adduct formation and anti-neoplastic effect. Free Radic. Biol. Med. *95*, 216–229.

34. Xiao, X., Yu, S., Li, S., Wu, J., Ma, R., Cao, H., Zhu, Y., and Feng, J. (2014). Exosomes: decreased sensitivity of lung cancer A549 cells to cisplatin. PLoS ONE *9*, e89534.

35. Krajcí, D., Mares, V., Lisá, V., Spanová, A., and Vorlícek, J. (2000). Ultrastructure of nuclei of cisplatin-treated C6 glioma cells undergoing apoptosis. Eur. J. Cell Biol. *79*, 365–376.

36. Köpf-Maier, P., and Mühlhausen, S.K. (1992). Changes in the cytoskeleton pattern of tumor cells by cisplatin in vitro. Chem. Biol. Interact. *82*, 295–316.

37. Zhang, F., Zhang, W., Liu, L., Fisher, C.L., Hui, D., Childs, S., Dorovini-Zis, K., and Ling, V. (2000). Characterization of ABCB9, an ATP binding cassette protein associated with lysosomes. J. Biol. Chem. *275*, 23287–23294.

38. Dong, Z., Zhong, Z., Yang, L., Wang, S., and Gong, Z. (2014). MicroRNA-31 inhibits cisplatin-induced apoptosis in non-small cell lung cancer cells by regulating the drug transporter ABCB9. Cancer Lett. *343*, 249–257.

39. Cekaite, L., Rantala, J.K., Bruun, J., Guriby, M., Ågesen, T.H., Danielsen, S.A., Lind, G.E., Nesbakken, A., Kallioniemi, O., Lothe, R.A., and Skotheim, R.I. (2012). miR-9, -31, and -182 deregulation promote proliferation and tumor cell survival in colon cancer. Neoplasia *14*, 868–879.

40. Hua, D., Ding, D., Han, X., Zhang, W., Zhao, N., Foltz, G., Lan, Q., Huang, Q., and Lin, B. (2012). Human miR-31 targets radixin and inhibits migration and invasion of glioma cells. Oncol. Rep. *27*, 700–706.

41. Stewart, D.J. (2007). Mechanisms of resistance to cisplatin and carboplatin. Crit. Rev. Oncol. Hematol. *63*, 12–31.

42. Kuusisto, H.V., and Jans, D.A. (2015). Hyper-dependence of breast cancer cell types on the nuclear transporter Importin β1. Biochim Biophys Acta. *1853*, 1870–1878.

43. Burger, H., Loos, W.J., Eechoute, K., Verweij, J., Mathijssen, R.H.J., and Wiemer, E.A.C. (2011). Drug transporters of platinum-based anticancer agents and their clinical significance. Drug Resist. Updat. *14*, 22–34.

44. Laurila, E., Vuorinen, E., Savinainen, K., Rauhala, H., and Kallioniemi, A. (2014). KPNA7, a nuclear transport receptor, promotes malignant properties of pancreatic cancer cells in vitro. Exp. Cell Res. *322*, 159–167.

45. Ouar, Z., Lacave, R., Bens, M., and Vandewalle, A. (1999). Mechanisms of altered sequestration and efflux of chemotherapeutic drugs by multidrug-resistant cells. Cell Biol. Toxicol. *15*, 91–100.

46. Boac, B.M., Xiong, Y., Marchion, D.C., Abbasi, F., Bush, S.H., Ramirez, I.J., Khulpateea, B.R., Clair McClung, E., Berry, A.L., Bou Zgheib, N., et al. (2016). Micro-RNAs associated with the evolution of ovarian cancer cisplatin resistance. Gynecol. Oncol. *140*, 259–263.

47. Pennati, M., Lopergolo, A., Profumo, V., De Cesare, M., Sbarra, S., Valdagni, R., Zaffaroni, N., Gandellini, P., and Folini, M. (2014). miR-205 impairs the autophagic flux and enhances cisplatin cytotoxicity in castration-resistant prostate cancer cells. Biochem. Pharmacol. *87*, 579–597.

48. Dehghanzadeh, R., Jadidi-Niaragh, F., Gharibi, T., and Yousefi, M. (2015). MicroRNA-induced drug resistance in gastric cancer. Biomed. Pharmacother. *74*, 191–199.

49. Drayton, R.M., Dudziec, E., Peter, S., Bertz, S., Hartmann, A., Bryant, H.E., and Catto, J.W. (2014). Reduced expression of microRNA-27a modulates cisplatin resistance in bladder cancer by targeting the cystine/glutamate exchanger SLC7A11. Clin. Cancer Res. *20*, 1990–2000.

50. Ohashi-Kobayashi, A., Ohashi, K., Du, W.B., Omote, H., Nakamoto, R., Al-Shawi, M., and Maeda, M. (2006). Examination of drug resistance activity of human TAP-like (ABCB9) expressed in yeast. Biochem. Biophys. Res. Commun. *343*, 597–601.

51. Öhrvik, H., and Thiele, D.J. (2015). The role of Ctr1 and Ctr2 in mammalian copper homeostasis and platinum-based chemotherapy. J. Trace Elem. Med. Biol. *31*, 178–182.

52. Zisowsky, J., Koegel, S., Leyers, S., Devarakonda, K., Kassack, M.U., Osmak, M., and Jaehde, U. (2007). Relevance of drug uptake and efflux for cisplatin sensitivity of tumor cells. Biochem. Pharmacol. *73*, 298–307.

53. Stordal, B., Hamon, M., McEneaney, V., Roche, S., Gillet, J.-P., O'Leary, J.J., Gottesman, M., and Clynes, M. (2012). Resistance to paclitaxel in a cisplatin-resistant ovarian cancer cell line is mediated by P-glycoprotein. PLoS ONE *7*, e40717.

54. Kalayda, G.V., Wagner, C.H., Buss, I., Reedijk, J., and Jaehde, U. (2008). Altered localisation of the copper efflux transporters ATP7A and ATP7B associated with cisplatin resistance in human ovarian carcinoma cells. BMC Cancer *8*, 175.

55. Zhang, Q., Ran, R., Zhang, L., Liu, Y., Mei, L., Zhang, Z., Gao, H., and He, Q. (2015). Simultaneous delivery of therapeutic antagomirs with paclitaxel for the management of metastatic tumors by a pH-responsive anti-microbial peptide-mediated liposomal delivery system. J. Control. Release *197*, 208–218.

56. Janssen, H.L., Reesink, H.W., Lawitz, E.J., Zeuzem, S., Rodriguez-Torres, M., Patel, K., van der Meer, A.J., Patick, A.K., Chen, A., Zhou, Y., et al. (2013). Treatment of HCV infection by targeting microRNA. N. Engl. J. Med. *368*, 1685–1694.

57. Kao, S.C., Fulham, M., Wong, K., Cooper, W., Brahmbhatt, H., MacDiarmid, J., Pattison, S., Sagong, J.O., Huynh, Y., Leslie, F., et al. (2015). A significant metabolic and radiological response after a novel targeted microRNA-based treatment approach in malignant pleural mesothelioma. Am. J. Respir. Crit. Care Med. *191*, 1467–1469.

58. Reid, G., Kao, S.C., Pavlakis, N., Brahmbhatt, H., MacDiarmid, J., Clarke, S., Boyer, M., and van Zandwijk, N. (2016). Clinical development of TargomiRs, a miRNA mimic-based treatment for patients with recurrent thoracic cancer. Epigenomics *8*, 1079–1085.

OMTN, Volume *8*

Supplemental Information

MicroRNA-31 Regulates Chemosensitivity in Malignant Pleural Mesothelioma

Hannah L. Moody, Michael J. Lind, and Stephen G. Maher

A

Cell line	Mean miR-31 CT value	Mean RNU-6 CT value
NCI-H2452 parent	38.96061	19.04827
NCI-H2452 miR-VC	38.85159	18.65891
NCI-H2452 miR-31	27.66615	19.33311
P31	24.86754	19.42408
NCI-H2052	23.28798	19.36644
MSTO-211H	27.50758	23.85113

B

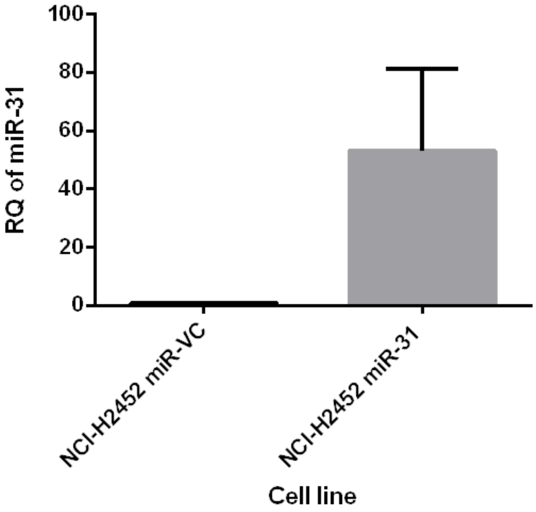

Supplementary Figure 1. MiR-31 status in MPM cell lines. (A) C_T values of MPM cell lines comparing miR-31 expression. (B) qPCR evaluating the relative level of expression miR-31 between vector control and miR-31 transfect cells over a period of 6 months (*n*=5). All qPCR runs were loaded with the maximum template of 100ng cDNA.

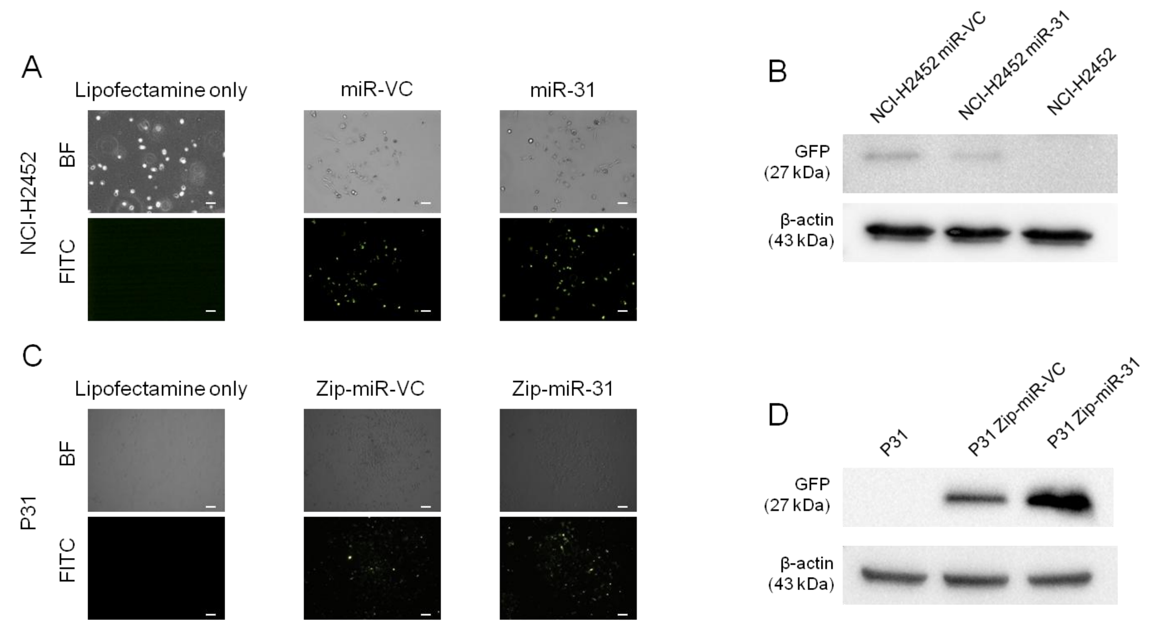

Supplementary Figure 2. Confirmation of stable transfection. (A) The NCI-H2452 cell line was stably transfected with corresponding overexpression vectors which had GFP reporter sequence. (B) Representative Western blot indicating GFP expression in transfected NCI-H2452 cell line. (C) The P31 cell line was stably transfected with corresponding silencing vectors which had GFP reporter sequence. (D) Representative Western blot indicating GFP expression in transfected P31 cell line. Images shown from FITC channel of Carl Zeiss Axio Vert.A1 microscope, captured with AxioCamIC with 0.5x Camera Adapter. The objective used was LD A-Plan 10x/0.25 Ph1.

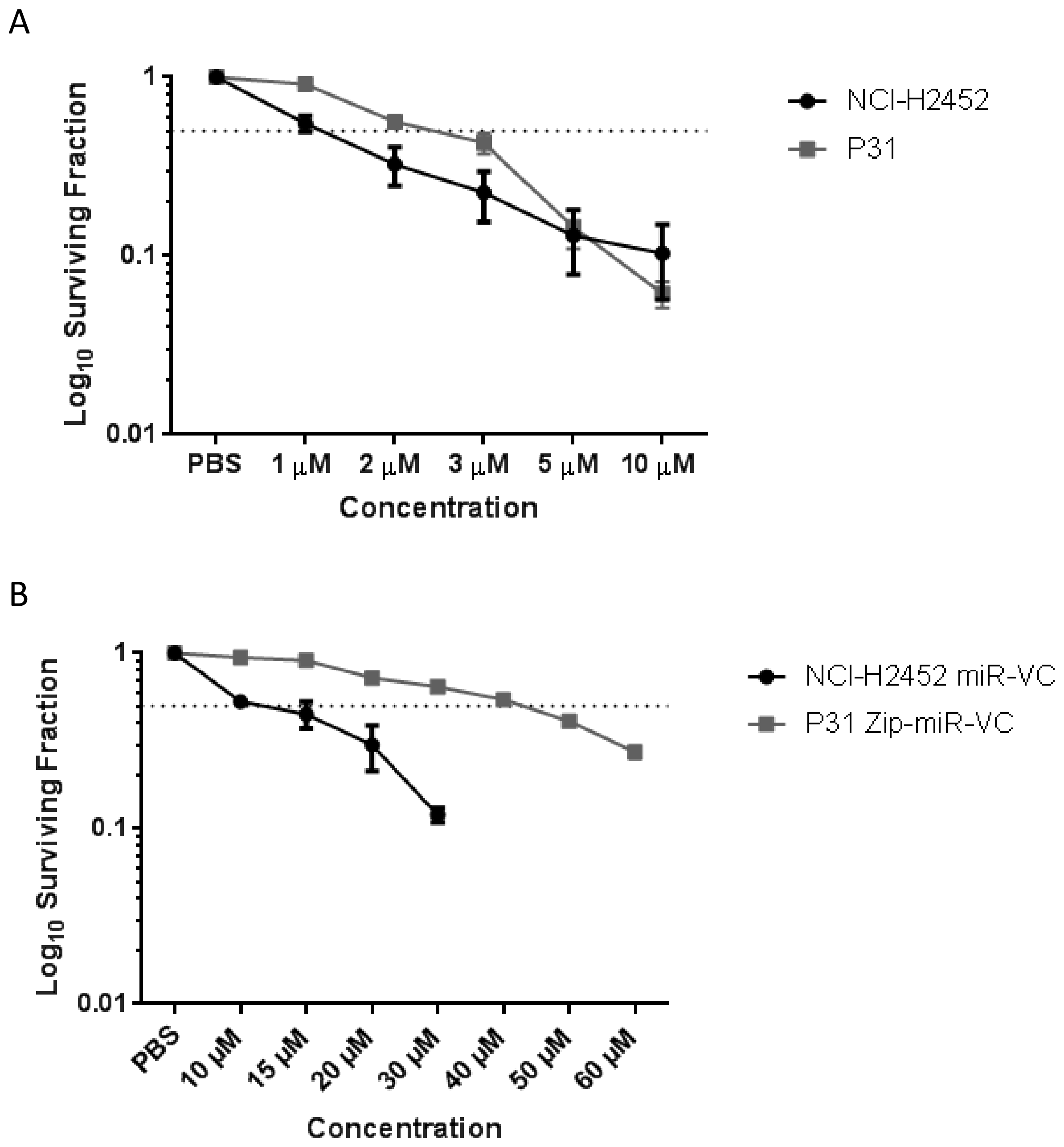

Supplementary Figure 3. IC_{50} doses of chemotherapeutics in MPM cell lines. (A) Dose response for cisplatin treatment for 24 h in the miR-31-deficient NCI-H2452 cell line (*n*=5) and P31 miR-31-positive cell line (*n*=3). Dose of 1 µM cisplatin determined for NCI-H2452, and 2 µM determined for P31. (B) Dose response for 48 h treatment with carboplatin in control transfected cell lines (*n*=3). Dose of 10 µM carboplatin determined for NCI-H2452 miR-VC, and 40 µM determined for P31 Zip-miR-VC. Cisplatin treatment was utilised for 24 h and carboplatin for 48 h in line with current literature, and due to more moderate effect of carboplatin treatment. All clonogenic assays were vehicle controlled with the analysis of PBS treated controls taken into account in surviving fraction calculations. Although plotted, where error bars are not visible, data replicates are within 0.05 surviving fraction.

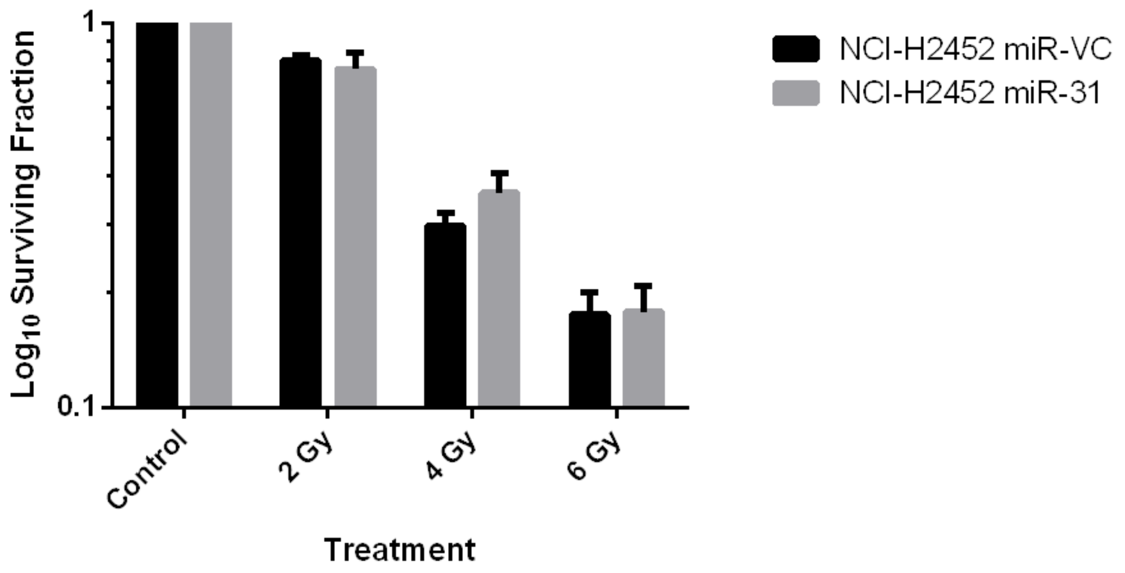

Supplementary Figure 4. The reintroduction of miR-31 does not modulate radiosensitivity in NCI-H2452. To determine the dose response for NCI-H2452 miR-VC and NCI-H2452 miR-31 cells, cells were irradiated with 2 Gy, 4 Gy and 6 Gy at a rate of 1.87 Gy/min. Survival was measured by clonogenic assay (*n*=3). Controls were mock irradiated. Data presented as ± SEM.

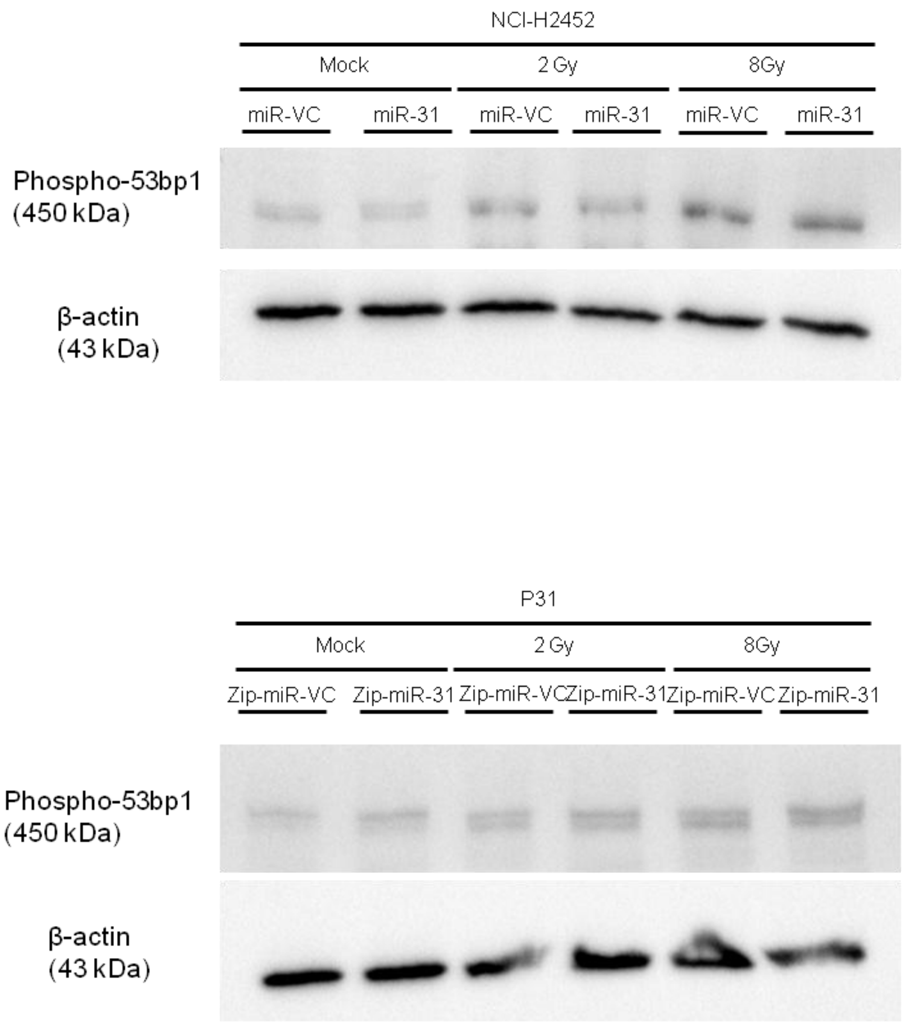

Supplementary Figure 5. MiR-31 does not modulate DNA damage induced by radiation treatment in MPM cells. Measuring phosphorylation of 53bp1 found no correlation between DNA damage induced by radiation treatment and miR-31 status. Cells were irradiated with 2 Gy and 8 Gy at a rate of 1.87 Gy/min. Controls were mock irradiated.

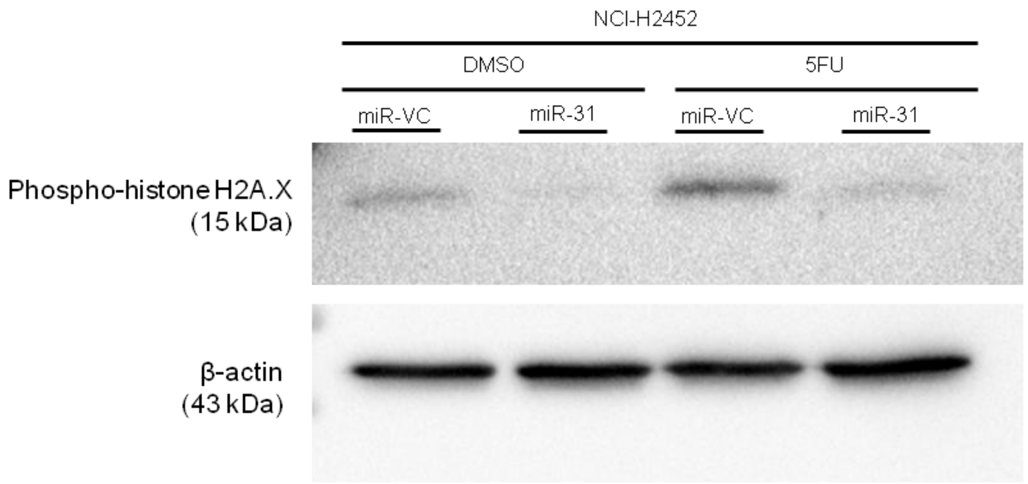

Supplementary Figure 6. Treatment with non-platinum based chemotherapeutics also decreases DNA damage. Treatment with 5FU induced minor DNA damage when miR-31 was reintroduced in the NCI-H2452 cell line, in accordance with cisplatin and carboplatin treatment. Cells were treated with 500µM 5FU for 24 hours.

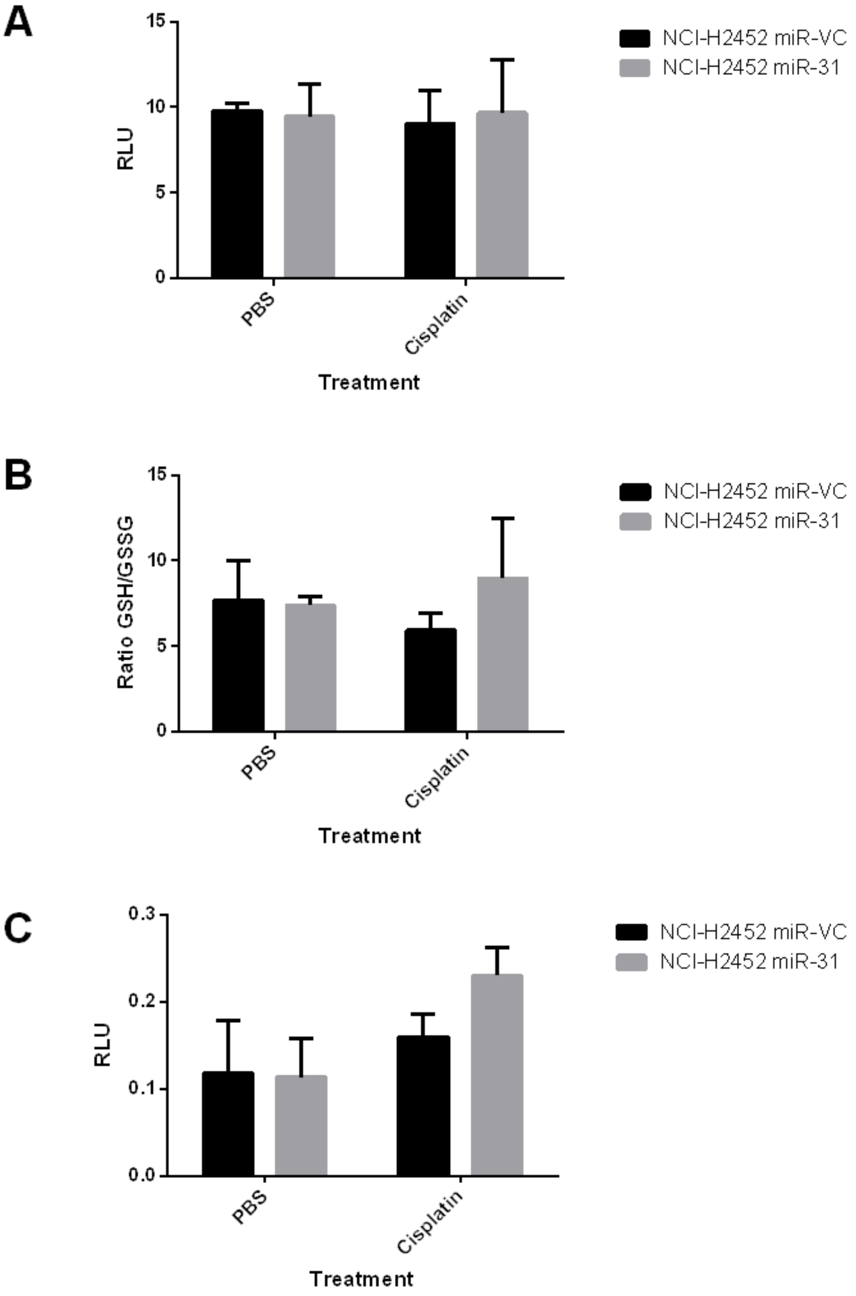

Supplementary Figure 7. Antioxidant and oxidant levels are negligibly altered by re-expression of miR-31. (A) Total glutathione levels were unaltered upon re-expression of miR-31, as measured by GSH/GSSG glo™ (Promega) assay (*n*=2). (B) Glutathione/Oxidised glutathione ratio was unaltered with miR-31 reintroduction, as measured by GSH/GSSG glo™ (Promega) assay (*n*=2). (C) Reactive oxygen species levels were moderately increased by re-expression of miR-31 with cisplatin treatment, as measured by ROS glo™ (Promega) assay (*n*=2). Data presented as ± SEM.

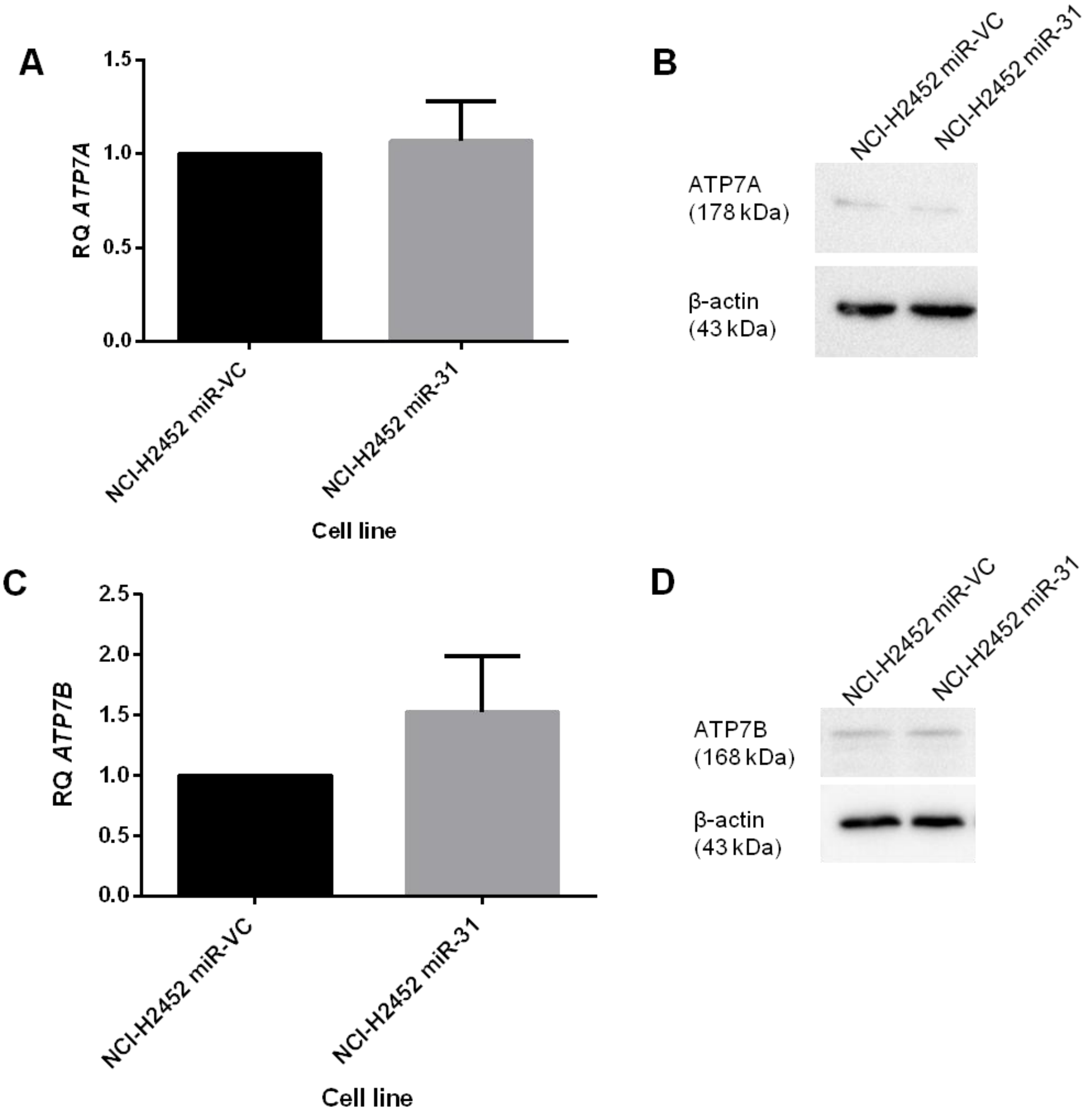

Supplementary Figure 8. MiR-31 rehabilitation in NCI-H2452 cells does not significantly alter the expression of copper efflux transporters. (A) Although a trend is apparent, there is no significant relative expression level of the transporter *ATP7A* in miR-31 compared to miR-VC equivalent (*n*=2). (B) ATP7A protein levels appear to be unaltered by miR-31 reintroduction. Data presented as the mean ± SEM. (C) Although a trend is apparent, there is no significant relative expression level of the transporter *ATP7B* in miR-31 compared to miR-VC equivalent (*n*=4). (D) ATP7B protein levels appear to be unaltered by miR-31 reintroduction. Data presented as the mean ± SEM.

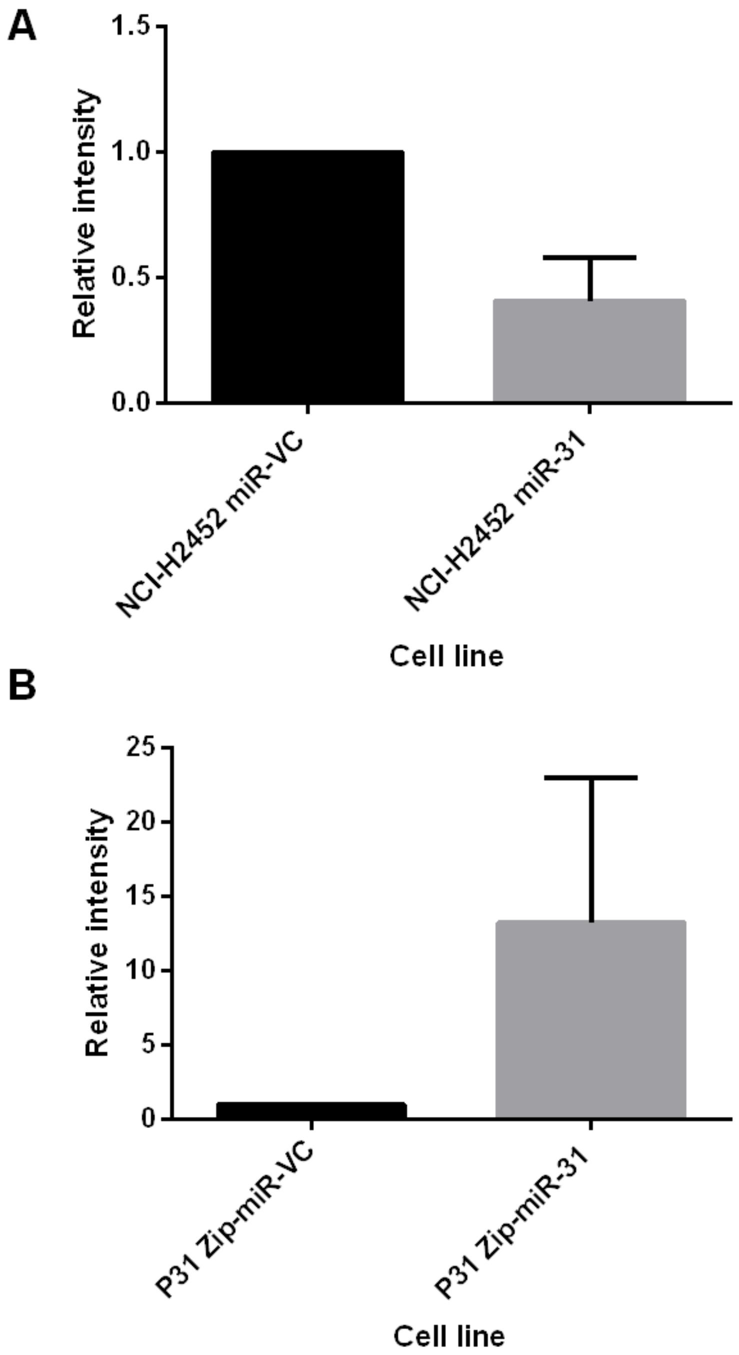

Supplementary Figure 9. Densitometry analysis for phospho-histone H2A.X. (A) There is a downregulation in the DNA damage marker phospho-histone H2A.X with miR-31 reintroduction, and an upregulation (B) with miR-31 suppression (*n*=3). Data presented as ± SEM.

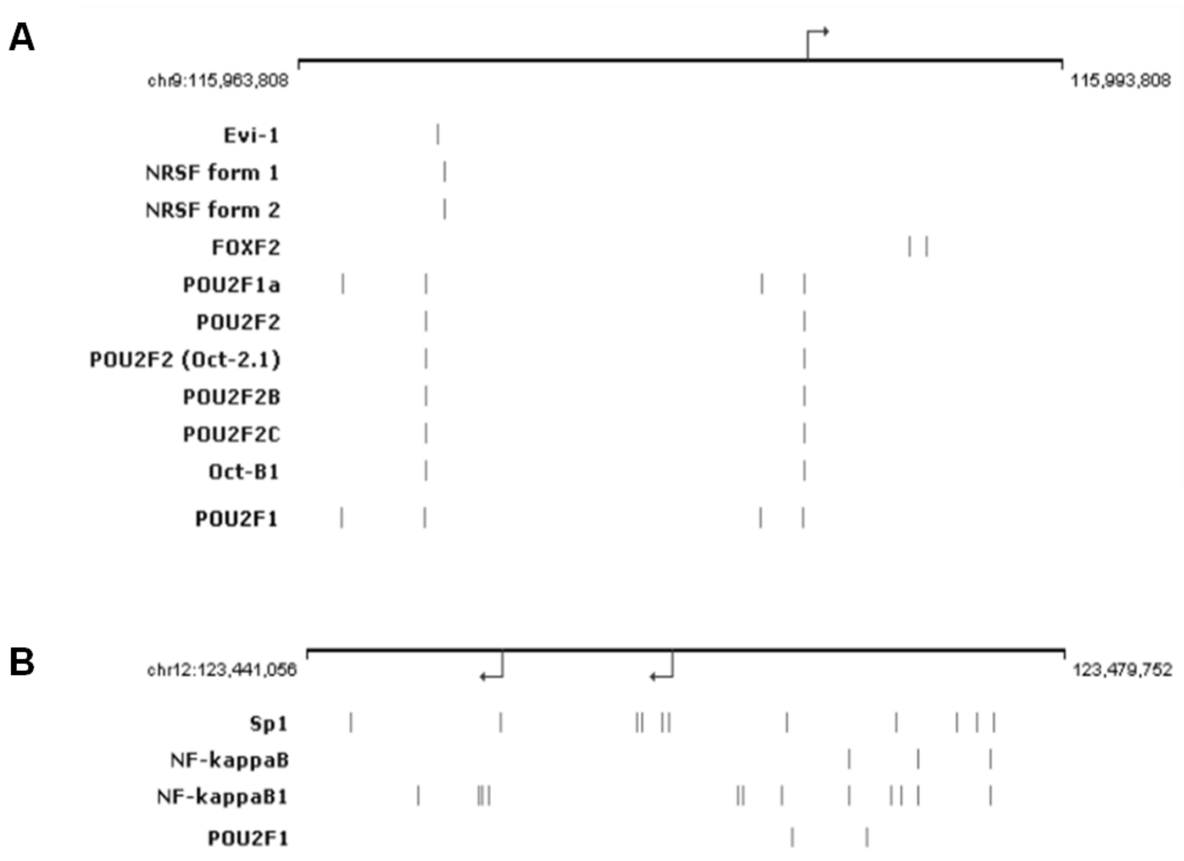

Supplementary Figure 10. Potential transcriptional regulators of genes *slc31a1* (CTR1) and *abcb9* (ABCB9). (A) Transcription factors regulating gene *slc31a1*. The image displays transcription factor binding sites in this gene promoter as predicted by SABiosciences' Text Mining Application and the UCSC Genome Browser. The *pou2f1* gene relates to the bipotential transcription factor OCT1. (B) Transcription factors regulating gene *abcb9*. The image displays transcription factor binding sites in this gene promoter as predicted by SABiosciences' Text Mining Application and the UCSC Genome Browser. The *pou2f1* gene relates to the bipotential transcription factor OCT1. (Adapted from http://www.sabiosciences.com/chipqpcrsearch.php).

Supplementary Table. 1. Target prediction for OCT1. Utilising bioinformatic tools, pairing of miR-31 with the *pou2f1* (OCT1) gene was performed. Of the approximately 190 predicted miRNA that may potentially bind to OCT1, miR-31 appears to be one of the most conserved. (Adapted from http://mircode.org/?gene=pou2f1&mirfam=&class=&cons=&trregion=, accessed 290615 and http://www.targetscan.org/cgibin/targetscan/vert_61/view_gene.cgi?taxid=9606&rs=NM_001198783&members=&showcnc=0&shownc=0&showncf=#miR-31, accessed 290615).

<table>
<tr><th rowspan="2">microRNA family</th><th rowspan="2">Predicted consequential pairing of target region (top) and miRNA (bottom)</th><th rowspan="2">Seed position</th><th rowspan="2">Transcript region</th><th colspan="3">Conservation</th></tr>
<tr><th>Primates</th><th>Mammals</th><th>Other vertebrates</th></tr>
<tr><td>miR-31</td><td>5' ...UUCUUAGUGAGAUCAUCUUGCCA...
|||||||
3' UCGAUACGGUCGUAGAACGGA</td><td>chr1:167389158</td><td>3pUTR</td><td>78%</td><td>61%</td><td>0%</td></tr>
<tr><td>miR-31</td><td>5' ...UUUUUCUUAUAUCUGUCUUGCCA...
|||||||
3' UCGAUACGGUCGUAGAACGGA</td><td>chr1: 167391236</td><td>3pUTR</td><td>100%</td><td>65%</td><td>23%</td></tr>
</table>

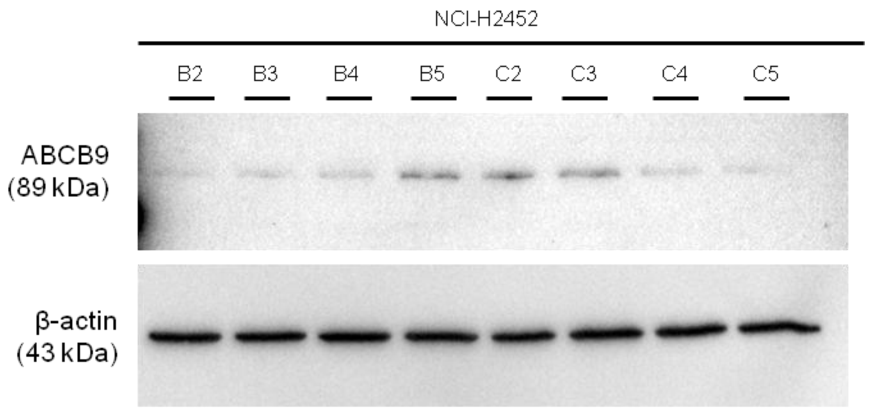

Supplementary Figure 11. ABCB9 overexpression is stable selected clones. NCI-H2452 miR-31-null cell line was transfected with EX-NEG vector control plasmid and an ABCB9 overexpression plasmid. Clonal populations were selected and grown under puromycin selection. Clones B2, B3, B4 and B5 correspond to EX-NEG vector control populations. Clones C2, C3, C4, C5 correspond to ABCB9 overexpressing clones. Clones C2 and C3 were selected as high expressing clones, C4 and C5 classified as low ABCB9 expressing clones.

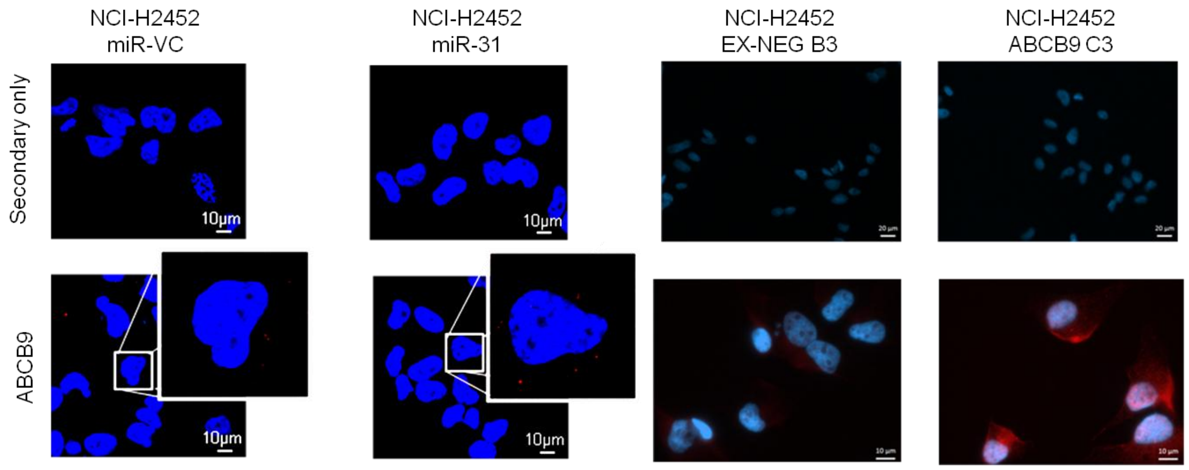

Supplementary Figure 12. ABCB9 overexpression in miR-31 reintroduced versus NCI-H2452 clonal variants. Immunoflourescent images showing overexpression of ABCB9 in transfected cells. ABCB9 staining (red), nuclei stained with DAPI (blue). Images captured on Zeiss Axio Vert.A1 with N-Achroplan 100x objective. Images for both EX-NEG and ABCB9 overexpressing populations are set at the same exposure in the same channels.
